# MAPK4 inhibits the early aberrant activation of B cells in rheumatoid arthritis by promoting the IRF4-SHIP1 signaling pathway

**DOI:** 10.1038/s41419-025-07352-2

**Published:** 2025-01-26

**Authors:** Pei Huang, Guangli Yang, Pingping Zhang, Yin Zhu, Yaning Guan, Jian Sun, Qian Li, Yang An, Xiaoqi Shi, Juanjuan Zhao, Chaohong Liu, Zhixu He, Yan Chen, Zuochen Du

**Affiliations:** 1 Department of Pediatrics, Affiliated Hospital of Zunyi Medical University, Zunyi, China; 2 Guizhou Children’s Hospital, Zunyi, China; 3 Collaborative Innovation Center for Tissue Injury Repair and Regenerative Medicine of Zunyi Medical University, Zunyi, China; 4 Department of Immunology, Zunyi Medical University, Zunyi, China; 5 Department of Pathogen Biology, Tongji Medical College, Huazhong University of Science and Technology, Wuhan, China

**Keywords:** B-cell receptor, Rheumatoid arthritis

## Abstract

The involvement of B lymphocytes in the pathogenesis of rheumatoid arthritis (RA) is well-established, with their early and aberrant activation being a crucial factor. However, the mechanisms underlying this abnormal activation in RA remain incompletely understood. In this study, we identified a significant reduction in MAPK4 expression in both RA patients and collagen-induced arthritis (CIA) mouse models, which correlates with disrupted B cell activation. Using MAPK4 knockout (KO) mice, we demonstrated that MAPK4 intrinsically promotes the differentiation of marginal zone (MZ) B cells. Loss of MAPK4 in KO mice enhances proximal BCR signaling and activates the PI3K-AKT-mTOR pathway, leading to heightened B cell proliferation. Notably, B cells from MAPK4 KO mice produce significantly higher levels of IL-6, a key pro-inflammatory cytokine in RA. Furthermore, MAPK4 KO mice exhibit impaired T cell-independent humoral immune responses. Mechanistically, MAPK4 inhibits the activation of the PI3K signaling pathway in B cells by activating the IRF4-SHIP1 pathway. Treatment with the MAPK4 agonist Vacquinol-1 enhances MZ B cell differentiation in WT mice and reduces IL-6 secretion in CIA mouse models. In summary, this study reveals the diverse roles of MAPK4 in regulating of B cell functions, with potential implications for developing therapeutic strategies for RA and related autoimmune diseases.

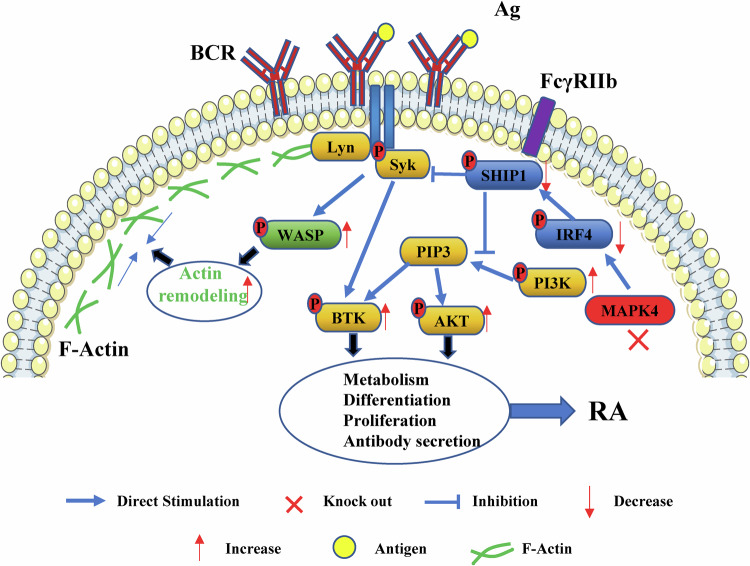

## Introduction

Rheumatoid Arthritis (RA) is characterized by painful, swollen joints and stiffness, which can lead to progressive joint damage and disability [1]. The importance of B cells in RA has been increasingly recognized, as they are involved not only in the production of autoantibodies, such as rheumatoid factor and anti-citrullinated protein antibodies, but also in antigen presentation, secretion of cytokines, and formation of ectopic lymphoid structures, all of which contribute to the chronic inflammatory process in the joints [2, 3]. B cell-targeted therapies, such as Rituximab, have significantly improved the treatment of RA by selectively depleting B cells, thereby reducing inflammation and slowing disease progression [4]. The recent development of newer agents like Atacicept, a broader and more comprehensive inhibitor of B cell maturation, have driven efforts to fine-tune and further optimize such B cell-targeted interventions [5]. Despite substantial progress, the precise mechanisms underlying the roles of B cells in RA remain incompletely understood.

Early B-cell activation, triggered by the binding of antigens to the B-cell receptor (BCR), initiates a highly organized cascade of signaling events that are critical for B-cell functions [6]. This process involves the formation of BCR micro clusters and the dynamic reorganization of cytoskeletons, which facilitates signal transductions and cell responses [7]. The connection between early activation and B cell functionality is well established. While tonic BCR signaling is essential for B cell survival, antigen-mediated BCR signaling primarily drives B cell proliferation and differentiation into plasma or memory cells [8]. The process plays an important role in adaptive immunity, and maintaining a delicate balance between effective immune responses and the prevention of the autoimmune reactions [9].

Markers of B-cell activation, including beta-2-microglobulin and B-cell activating factor of tumor necrosis factor family (BAFF), are significantly elevated in the serum of early RA patients compared to those with undifferentiated arthritis [10, 11]. These increased markers, which correlate with disease activity, rheumatoid factors levels, and anti-cyclic citrullinated peptide secretion, suggest that abnormal early activation of B cells may present in the circulation of patients with RA [12]. Such findings support a novel perspective on the early pathogenic role of B lymphocytes in RA and postulate that therapeutically targeting B cells may be effective in early-stage RA patients [13]. Despite substantial advancements in understanding BCR signaling activation in RA, the underlying mechanisms remain only partially elucidated. Abnormalities in the phosphorylation of key signaling molecules, such as Syk, BTK, and CD19 has been observed [14], along with disruptions in related signaling pathways [15]. However, the precise mechanisms responsible for these abnormalities are not yet fully understood, warranting further investigation.

MAPK4 (Mitogen-Activated Protein Kinase 4) is an atypical member of the MAPK family, primarily acting through phosphorylation of target proteins to regulate various biological processes, including cell proliferation, differentiation, and survival [16]. It also plays a crucial role in cancer, with its overexpression associated with poor prognosis in multiple cancers, including breast, thyroid, gliomas, prostate, and cervical cancers. MAPK4 promotes tumor cell proliferation, migration, survival, and drug resistance through several signaling pathways, such as AKT-mTOR, GATA2-AR, and PDK1. Given its pivotal role in cancer progression, MAPK4 represents a promising target for both diagnostic and therapeutic interventions [17].

In the immune system, MAPK4 regulates immune cell signaling and inflammatory responses, influencing both innate and adaptive immune reactions, and is involved in various inflammatory diseases [18, 19]. MAPK4 also participates in the activation of T cell signaling, promoting acute lung injury [20]. However, limited research has explored the role of MAPK4 in B cells, as most studies have focused on the influence of typical MAPK family members on immune cell signaling. In consideration of the involvement of MAPK pathways in cellular responses to a number of extracellular stimuli, it can be postulated that MAPK4 may play an important role in B cell activity and function.

In this study, we firstly identified the expression of MAPK4 and the early activation of B cells both in the RA patients and collagen-induced arthritis (CIA) mouse models. Subsequently, using MAPK4 knockout (KO) and wild-type (WT) mice, we validated the function of MAPK4 in the regulation of B cell development, differentiation, and early activation by flow cytometry, confocal microscopy, western blotting and other techniques. Our results confirmed that MAPK4 regulates B cell activation and function by promoting the activation of the IRF4-SHIP1 axis, which subsequently suppresses the phosphatidylinositol-3-kinase (PI3K) signaling. Importantly, an agonist of MAPK4 was shown to suppress IL-6 secretion in the CIA mouse model. Our study provides a potential strategy of targeted therapy in RA.

## Materials and Methods

### Mice

MAPK4 KO mice on a C57BL/6 background were generously provided by Professor Lin Xu from Zunyi Medical University, while C57BL/6 WT mice served as controls. DBA/1 mice were purchased from Vital River Laboratory Animal Technology Co., Ltd. (Beijing, China). All mice were housed under individually ventilated cage conditions at the Affiliated Hospital of Zunyi Medical University. All experimental protocols involving animals were conducted in accordance with the guidelines for the Care and Use of Laboratory Animals and were approved by the Laboratory Animal Welfare & Ethics Committee of Zunyi Medical University (ZMU21-2302-043).

### Patients

Eight newly diagnosed patients with RA and age-matched healthy control individuals were enrolled in this study. Informed consent was obtained from all participants. The study adhered to the principles outlined in the Declaration of Helsinki and received approval from the Ethics Committee of the Affiliated Hospital of Zunyi Medical University (KLLY-2024-075).

### Cell culture

B cells were isolated as previously reported [21]. The isolated cells were cultured in RPMI 1640 medium supplemented with 10% FBS (Gibco) at 37 °C with 5% CO_2_.

### Treatment with MAPK4 agonist Vacquinol-1 in vivo and in vitro

For in vitro stimulation, B cells were pretreated with 15 μM of the MAPK4 agonist Vacquinol-1 (5428-80-8, TargetMol) for 1 h before stimulation. For in vivo stimulation, WT mice, aged 6–8 weeks with a 1:1 male-to-female ratio, were randomly assigned to either the agonist group or the control group. Mice in the agonist group were administered intraperitoneal injections of 15 μM Vacquinol-1 every other day for 14 days. Mice in the control group were injected with an equal volume of PBS at the same time points.

### Flow cytometry and phos flow

For the analysis of B cell subset in RA patients, PBMCs were stained with antibodies including PerCP-anti-CD3, APC-anti-CD19, Percp/cy5.5-anti-CD19, PB-anti-CD38, PE/CY7-anti-CD27, BV510-anti-IgD, APC-anti-IgM, PE-anti-CD21. For detection of MAPK4 expression, PBMCs from HC and RA patients were stained with PerCP-anti-CD3 and APC-anti-CD19 antibodies. After fixation and permeabilization using the Foxp3 Staining Buffer Set (00-5523-00; eBioscience), the cells were labeled with anti-MAPK4 antibody (bs-1319R; Bioss) and Alexa Fluor™ 488 conjugated donkey anti-rabbit IgG (H + L) (A21206; life technologies), then analyzed by flow cytometry.

For the analysis of B cell in WT and MAPK4 KO mice, bone marrow cells were stained with antibodies including APC-anti-CD43, PE-anti-BP-1, PE/CY7-anti-CD24, BV421-anti-IgM, BV510-anti-B220 and 7AAD. Splenic lymphocytes were stained with FITC-anti-CD19, PE-anti-CD23, Percp-anti-IgD, APC-anti-CD21, PE/CY7-anti-Ki67, APC-anti-GL7, FITC-anti-CD95, and FITC-Annexin V. For B cell intracellular cytokine staining, B cells from WT or MAPK4 KO mice were isolated and stimulated with different conditions: CPG + anti-CD40 for IL-6 detection, CPG alone for IL-10 detection, and PMA + Ionomycin for IFN-γ detection at 37 °C for 5 h. After stimulation, cells were surface-stained with BV510-anti-B220, followed by fixation and permeabilization. The cells were then stained with PE-anti-IL-6, FITC-anti-IL-10, and PE/CY7-anti-IFN-γ antibodies. B1 cells collected from the peritoneal cavity were stained with antibodies including FITC-anti-CD19, PE-anti-CD5, Percp-anti-IgD, APC-anti-CD11b, BV421-anti-IgM.

For phospho-flow analysis, isolated PBMCs or splenic lymphocytes were labeled with APC-anti-CD19 or BV510-anti-B220 antibodies, respectively. Subsequently, the cells were activated using a previously described method [22]. Briefly, cells were incubated with soluble antigen (sAg) comprising Biotin-SP AffiniPure F(ab’)2 Fragment Goat Anti-Human IgG + IgM (H + L) (109-006-127; Jackson) or Alexa Fluor® 594-conjugated AffiniPure F(ab)2 Fragment Goat Anti-Mouse IgG + IgM (H + L) (115-586-068; Jackson) for 30 min, followed by streptavidin (016-000-114; Jackson) for 10 min on ice. BCR signaling activation was then induced at 37 °C for varying time intervals. After fixation, the cells were stained with antibodies specific for phosphorylated tyrosine residues, Alexafluor™ 488 phalloidin, pY, pBTK, BCR, ACTIN, pPI3K(p85), or pWASP. Secondary antibody AF405-conjugated goat anti-rabbit IgG (A31556; Lifetechnology) or Alexa Fluor® 488-conjugated AffiniPure Donkey Anti-Mouse IgG (H + L) (715-545-150, Jackson) was used for detection.

For T cell subpopulation analysis in MAPK4 KO mice, lymphocytes from the thymus, spleen, and lymph nodes were stained with antibodies including Percp-anti-CD44, APC-anti-CD25, APC/CY7-anti-TCRβ, PE/CY7-anti-CD62L, Pacblue-anti-CD4, BV510-anti-CD8, FITC-anti-Foxp3, PE/CY7-anti-Ki67, FITC-anti-IL2. For T cell intracellular cytokine staining, lymphocytes were stimulated for 5 h. at 37 °C, 5% CO_2_, in the presence of PMA + Ionomycin in combination with GolgiStop (55472; BD Pharmingen). After stimulation, the cells were surface-stained with PE anti-CD4, Percp-anti-CD44 and 7AAD, followed by fixation and permeabilization. The cells were then stained with APC-anti-IL4, PE/CY7-anti-IFN-γ, BV421-anti-IL17.

Subsequently, the stained cells were analyzed using a BD FACS Canto Plus flow cytometer, and data were further processed with FlowJo V10 software (Tree Star). The detailed information for the above-mentioned antibodies is provided in Table S1.

### Ex vivo antigen presentation assay

A total of 1 × 10^6^ B cells from WT or MAPK4 KO mice were isolated and incubated with 100 µL of 20 µg/mL Eα52–68 peptide (amino acid sequence: ASFEAQGALANIAVDKA; 13-5741-82, Thermo) at 37 °C for 1 h. The cells were then stained on ice with Biotin-Y-Ae antibody (1:400, Invitrogen, 13-5741-85), PE-SA, and FITC-B220. Finally, the stained cells were analyzed using flow cytometry.

### Western blot

Splenic B cells were stimulated with sAg, and the cell lysates were subjected to SDS-PAGE and subsequently transferred onto a PVDF membrane (Millipore, Germany). The membrane was then blocked with a 5% non-fat milk solution before probed with the following primary antibodies: anti-MAPK4, anti-pBTK, anti-BTK, anti-phosphotyrosine, anti-pPI3K(p85), anti-PI3K, anti-pAKT, anti-AKT, anti-pIKKβ, anti-IKKβ, anti-p-mTOR, anti-mTOR, anti-pS6, anti-S6, anti-pFOXO1, anti-FOXO1, anti-pSyk, anti-Syk, anti-pCD19, anti-CD19, anti-pSHIP1, anti-SHIP1, anti-AID, anti-pWASP, anti-pIRF4, and anti-β-ACTIN or anti-GAPDH, which were used as loading controls. Following incubation with HRP-linked secondary antibodies and subsequent washing steps, the blots were visualized using an imaging system (ChemiDoc™ Touch, BIO-RAD). The detailed information for the above-mentioned antibodies is provided in Table S1.

### Confocal microscopy

Splenic B cells from WT and MAPK4 KO mice were purified and subsequently incubated with anti-mouse CD16/CD32 to block Fc receptors. Following stimulation as previously described [21], cells were incubated with AF594-AffiniPure F(ab’)2 Fragment Goat Anti-Mouse IgG + IgM (H + L) (115-586-068; Jackson) and PE-streptavidin (405204; BioLegend) on ice for 30 and 10 min, respectively. BCR signaling was then stimulated at 37 °C for varying time intervals. After fixation and permeabilization, cells were stained with the following antibodies: anti-pBTK (87457S; Cell Signaling Technology), anti-phosphotyrosine (9411S; Cell Signaling Technology), anti-pPI3K(p85) (17366S; Cell Signaling Technology), anti-pWASP (AF7304; Affinity) and Phalloidin-AF488 (R37110; Lifetechnology). Confocal imaging was subsequently performed using a Leica STELLARIS 5 microscope, and data analysis was conducted using the Leica Application Suite X software (Leica, Germany).

### Rheumatoid arthritis model

CIA mouse model was established according to previous reports [23]. Male DBA/1 mice aged 8–10 weeks were randomly divided into a model group and a control group. The model group mice were injected with 0.1 mL of collagen (20011, Chondrex) and complete Freund adjuvant (7002, Chondrex) emulsion (1:1) on day 1, followed by a similar injection of collagen and incomplete Freund adjuvant (7002, Chondrex) emulsion (1:1) on day 21. The control group mice were injected with an equal volume of PBS at the same time points. Arthritis manifestation was evaluated every 2 days. On day 33, the mice were euthanized, and splenic B cells were isolated for subsequent flow cytometry and WB analysis.

### CO-immunoprecipitation

CO-immunoprecipitation experiments were conducted using a Classic Magnetic Protein A/G IP/Co-IP kit (YJ201; Epizyme) following the manufacturer’s instructions. Briefly, splenic B cell lysates from WT mice were prepared and incubated with anti-IRF4 (DF6198; Affinity), anti-MAPK4 (BS-1319R; Bioss) antibodies, or control IgG overnight at 4 °C, followed by an incubation with Protein A/G-agarose beads for an additional hour. After washing, IRF4 and MAPK4 were measured in all immunoprecipitation (IP) and input samples by Western blotting.

### Ca^2+^ flow

Purified splenic B cells from WT and MAPK4 KO mice were suspended in Ca^2+^-free HBSS (14175079; Gibco™) containing a final concentration of 0.5 μM calcium-sensitive dye Fluo-4 AM (273221-67-3; MedChemExpress) and incubate for 25 min at 37 °C. Subsequently, the cells were incubated with an BV510-anti-mouse/human CD45R/B220 antibody (103248; BioLegend) for 30 min. Following an initial 30 s recording on a flow cytometer, the cells were briefly mixed with preheated Biotin-SP-AffiniPure F(ab’)2 Fragment Goat Anti-Mouse IgG + IgM (H + L) (115-066-068; Jackson) to a final concentration of 10 μg/mL, and the recording continued for additional 5 min.

### B-Cell proliferation

B-Cell proliferation detection was performed as previously described [24]. Purified B cells were labeled with CFSE (HY-D0938; MedChemExpress) and stimulated with 5 μg/ml LPS (L2880; Sigma) or 10 μg/ml ODN1826 (tlrl-1826-1; InvivoGen) in the culture medium for 4 days. Then the cells were subjected to flow cytometry analysis after staining with BV510-B220 (103248; BioLegend) and 7-AAD (00-6993-50; ebioscience) on ice.

### Immunization and ELISA

WT and MAPK4 KO mice were intraperitoneally injected with 40 μg NP-KLH (N-5060-5; Biosearch) or 100 μg NP-FICOLL (sc-396292; SantaCruz) in 200 μL of PBS. After 2 weeks, the splenic lymphocytes were isolated for flow cytometry analysis. Additionally, the NP-specific levels of IgM and IgG_1_ in serum were determined by ELISA using NP-BSA (N-5050H-10; Biosearch)–coated plates, followed by incubation with anti-IgM (FNSA-0091; Proteintech) and anti-IgG1(FNSA-0086; Proteintech) secondary antibodies. For the detection of anti-double-stranded DNA (dsDNA) antibodies in serum, samples were collected from WT and MAPK4 KO mice, and the levels of anti-dsDNA antibodies were measured using a commercial ELISA kit (CSB-E11194, CUSABIO), following the manufacturer’s instructions.

### Bone marrow chimera

Bone marrow cells were extracted from femurs of 8 week-old CD45.2 WT/MAPK4 KO and CD45.1 mice with a 1:1 male-to-female ratio. Bone marrow cell mixtures at a 1:1 ratio of CD45.1 and CD45.2 WT or MAPK4 KO were prepared and randomly intravenously injected into 12–15 week-old CD45.1 mice that had been sublethally irradiated (6 Gy). After 8 weeks, bone marrow and splenic lymphocytes were collected for flow cytometric analysis [25].

### BCR internalization

Splenic B cells from WT and MAPK4 KO mice were incubated with BV510-anti-B220 (103248; BioLegend) on ice for 30 min, followed by incubation with Biotin-SP-AffiniPure F(ab’)_2_ Fragment Goat Anti-Mouse IgG + IgM (H + L) (115-066-068; Jackson) and PE-Streptavidin (405204; BioLegend) on ice for an additional 30 min. Subsequently, the cells were incubated at 37 °C for 0, 2, 5, 10 or 20 min, then fixed with PFA (final concentration 4%, WI333638, Thermo Fisher Scientific) on ice for 30 min and subjected to flow cytometric analysis. BCR internalization was assessed by comparing the mean fluorescence intensity (MFI) of biotin-conjugated F(ab’)_2_ Ig-labeled BCR proteins remaining on the cell surface between B cells from WT and MAPK4 KO mice.

### H&E staining

The spleens of WT and MAPK4 KO mice were fixed with 4% PFA at room temperature. The tissues were then dehydrated through a gradient of ethanol and embedded in paraffin. Sections of 4 μm were sliced with a paraffin slicer. The slices were then deparaffinized and stained with hematoxylin and eosin. Images were captured using an optical microscope.

### Immunofluorescence

The spleens from WT and MAPK4 KO mice were embedded in OCT, frozen in liquid nitrogen, and stored at −80 °C. The frozen samples were sectioned at 5 µm using a cryostat, fixed with acetone for 5 min, air-dried, and stored at −80 °C for subsequent immunofluorescence staining. Sections were incubated with 5% BSA and biotin-IgM (1:50, 13-5790-8; Invitrogen), FITC-CD169 (MA5-28189; Invitrogen), and anti-mouse APC-IgD (17-5993-82; Invitrogen) overnight at 4 °C, followed by incubated with PE-Streptavidin (1:200; 405204; BioLegend). The slides were then mounted after washing with an imaging antifading reagent to prevent fluorescence quenching. Images were captured and analyzed using a confocal microscope (Leica).

### Statistical analysis

The number of animals or samples and the number of experimental replicates are specified in the figure legends, with the sample size determined based on previous experience and preliminary data. Two-tailed unpaired *t*-test and One-way ANOVA were performed using the GraphPad Prism software version 8.0.1 for Windows (San Diego, California USA). A significance level of *P* < 0.05 was considered indicative of a statistically significant difference between groups.

## Results

### Reduced expression of MAPK4 in B Cells of RA patients and CIA mouse models

**Fig. 1:**
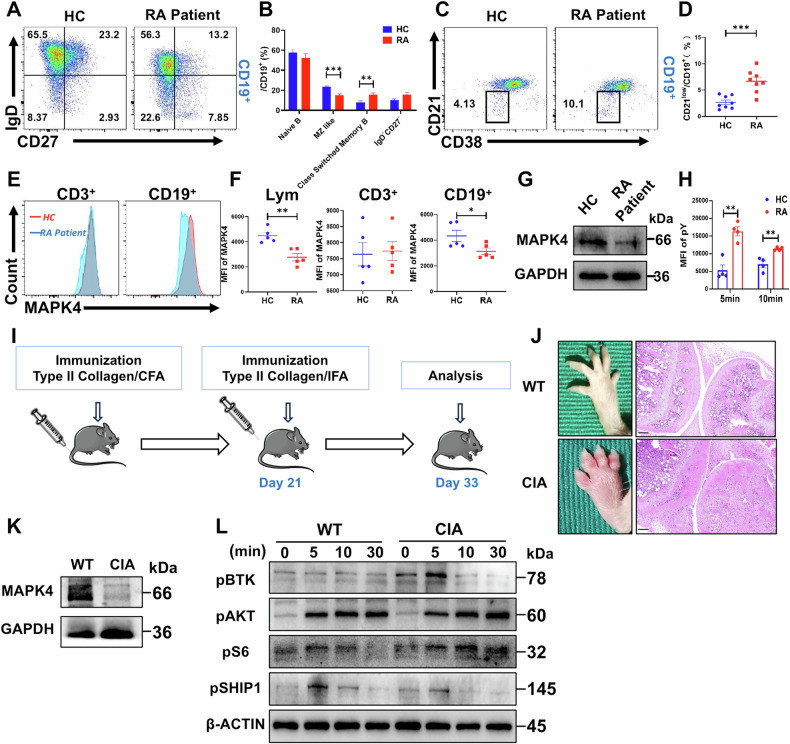
Reduced expression of MAPK4 in B Cells of Rheumatoid Arthritis patients and mouse models. **A**, **B** Flow cytometric analysis of B cell subsets in HCs and newly diagnosed RA patients (*n* = 8). **C**, **D** Proportion of CD21^low^ cells in B cells from HCs and newly diagnosed RA patients (*n* = 8). **E**, **F** MAPK4 expression detected by flow cytometry in lymphocytes, CD3^+^ T cells, and CD19^+^ B cells from HCs and newly diagnosed RA patients (*n* = 5). **G** Western blot analysis of MAPK4 expression in B cells from HCs and newly diagnosed RA patients. **H** Comparison of MFI of pY between RA patients and HCs after in vitro stimulation of peripheral blood B cells with sAg at different time points (*n* = 4). **I** Schematic illustration of the development of a CIA mouse model. **J** Images of paws and H&E staining of knee joints from WT and CIA mice. Scale bar = 100 μm. **K** Western Blot analysis of MAPK4 expression in B cells from WT and CIA mice. **L** Western blot analysis was performed to evaluate the levels of pBTK, pAKT, pS6, and pSHIP1 in B cells from WT and CIA mice following in vitro stimulation with sAg at different time points, using β-ACTIN as a control. The data are presented as mean ± SEM. Representative blots from three independent experiments are shown. *P < 0.05; **P < 0.01; ***P < 0.001.

To assess MAPK4 expression in RA patients, we initially examined changes in B cell subsets in eight newly diagnosed RA patients using flow cytometry. RA patients exhibited a decreased proportion of marginal zone (MZ) like B cells (CD27^+^IgD^+^) and an increased proportion of class-switched B cells (CD27^+^IgD^-^) compared to healthy controls (Fig. 1A, B). Additionally, the proportion of CD21^low^ cells was elevated in RA patients (Fig. 1C, D), consistent with reports in the literature [26, 27]. Further examination via flow cytometry revealed reduced MAPK4 expression in B cells from RA patients (Fig. 1E, F). This was confirmed by sorting B cells and detecting MAPK4 expression using Western Blot analysis, which showed decreased MAPK4 expression in RA patient B cells (Fig. 1G). Furthermore, we stimulated peripheral blood B cells with sAg in vitro and observed elevated phosphorylated tyrosine (pY) levels in RA patients compared to healthy controls (Fig. 1H), indicating aberrant early activation of B cells in RA patients.

To investigate MAPK4’s involvement in B cell regulation in RA mice, we developed a CIA mouse model (Fig. 1I). Initial confirmation included joint swelling and increased infiltration of inflammatory cells in the CIA mouse model using HE staining (Fig. 1J). Examination of MAPK4 protein expression in B cells within this model revealed decreased expression in CIA mice compared to normal controls (Fig. 1K). Additionally, in vitro stimulation of B cells with sAg showed increased levels of early activation signaling molecules (pBTK, pAKT, and pS6) but decreased levels of pSHIP1 in CIA mice compared to WT mice (Fig. 1L). These findings suggest a reduced expression of MAPK4 in B cells, along with early abnormal activation of B cells in both RA patients and CIA mice.

### MAPK4 promotes the differentiation of MZ B cells through a B cell-intrinsic mechanism

**Fig. 2:**
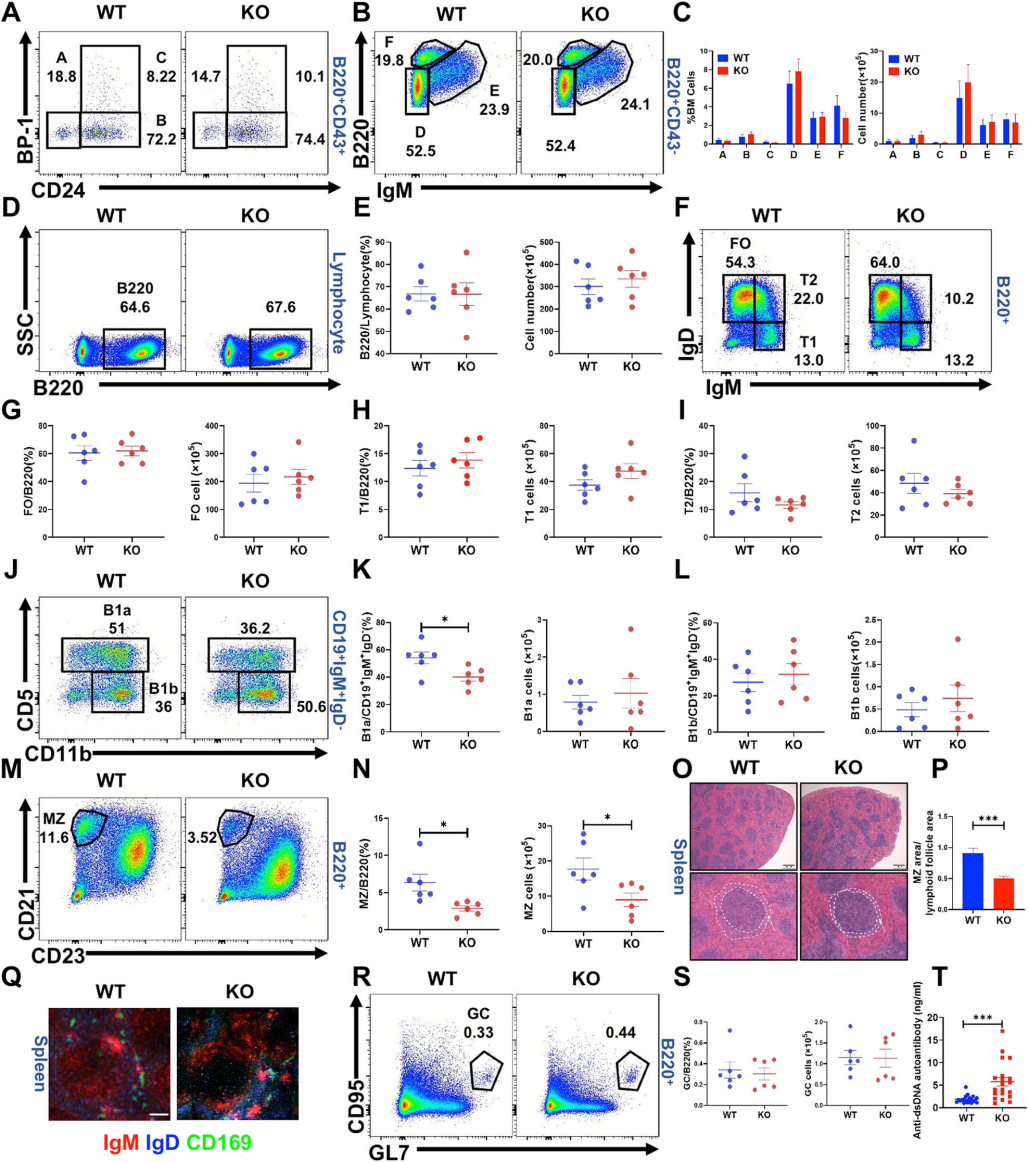
MAPK4 promotes the differentiation of marginal zone (MZ) B cells. **A**–**C** Flow cytometric analysis of the percentages and absolute numbers of bone marrow B cell subpopulations in WT and MAPK4 KO mice, including pre-pro-B cells (A), pro-B cells (B), early pre-B cells (C), late pre-B cells (D), immature B cells (E), and recirculating B (F) cells (*n* = 6). **D**, **E** Flow cytometric analysis of proportions and absolute numbers of splenic B cells in WT and MAPK4 KO mice (*n* = 6). **F**–**N** Flow cytometric analysis of peripheral B cell subpopulations in WT and MAPK4 KO mice (*n* = 6). The proportions and absolute numbers of FO (**F**, **G**), T1 (**F**, **H**), T2 (**F**, **I**), B1a (**J**, **K**), B1b (**J**, **L**) and MZ (**M**, **N**) cells were compared between WT and MAPK4 KO mice. **O**-**P** Proportion of MZ area in the lymphoid follicle area, analyzed by H&E staining of the spleen (*n* = 16). Scale bar = 200 μm. **Q** Immunofluorescence staining of spleen MZ cells from WT and MAPK4 KO mice. Scale bar = 25 μm. **R**, **S** Proportions and absolute numbers of splenic GC B cells under unimmunized conditions were detected and compared between WT and MAPK4 KO mice (*n* = 6). **T** Serum anti-dsDNA antibodies titers in WT and MAPK4 KO mice (*n* = 20). All data are presented as mean ± SEM. *P < 0.05; **P < 0.01; ***P < 0.001.

We utilized MAPK4 KO mice to further investigate the impact of MAPK4 on B cells. We initially assessed MAPK4 expression in splenic B cells through Western blotting. The results revealed that MAPK4 expression was undetectable in B cells from MAPK4 KO mice compared to age-matched WT controls (Fig. S1A). Subsequently, we analyzed B cell subpopulations in MAPK4 KO mice using flow cytometry. There were no significant differences in bone marrow B cell subpopulations between WT and MAPK4 KO mice (Fig. 2A–C). Further examination of peripheral B cell subpopulations revealed no significant differences in total B, follicular (FO), transitional 1 (T1), and transitional 2 (T2) cells between WT and MAPK4 KO mice (Fig. 2D–I). Although the percentage of B1a cells was reduced in MAPK4 KO mice, their absolute cell number remained unchanged. In contrast, the percentage of B1b cells showed no difference (Fig. 2J–L). Of note, there was a significant reduction in both the proportion and absolute number of MZ cells in MAPK4 KO mice (Fig. 2M, N). H&E staining further confirmed a significant reduction in the MZ area in MAPK4 KO mice compared to WT mice (Fig. 2O, P). Immunofluorescence staining for MZ cells revealed a decreased fluorescence intensity in MAPK4 KO mice (Fig. 2Q). Moreover, examination of splenic germinal center (GC) B cells under unimmunized conditions showed no significant changes between WT and MAPK4 KO mice (Fig. 2R-S). Additionally, we measured the titers of anti-double-stranded DNA (anti-dsDNA) antibodies in the serum of WT and MAPK4 KO mice, and found elevated concentrations of anti-dsDNA antibodies in MAPK4 KO mice (Fig. 2T).

To further demonstrate the regulatory role of MAPK4 in MZ B cell differentiation, bone marrow chimera mice were generated by transferring BM cells from WT or MAPK4 KO mice into recipient mice. Analysis showed a lower proportion of MZ B cells in MAPK4 KO BM chimera mice compared to WT BM chimera mice, with no significant changes in the proportions of other subpopulations, except for an increase in the numbers of B220^+^ and FO cells (Fig. S1B–P).

Overall, these findings suggest that MAPK4 positively regulates MZ B cell development in the mouse spleen via a B cell-intrinsic manner. What’s more, the Annexin V and Ki67 positive cell rates in B cell subsets showed no significant differences between the two groups (Fig. S2A–D).

### MAPK deficiency leads to decreased differentiation of thymus and spleen CD4 and CD8 cells, with no significant impact on T cell homeostasis

**Fig. 3:**
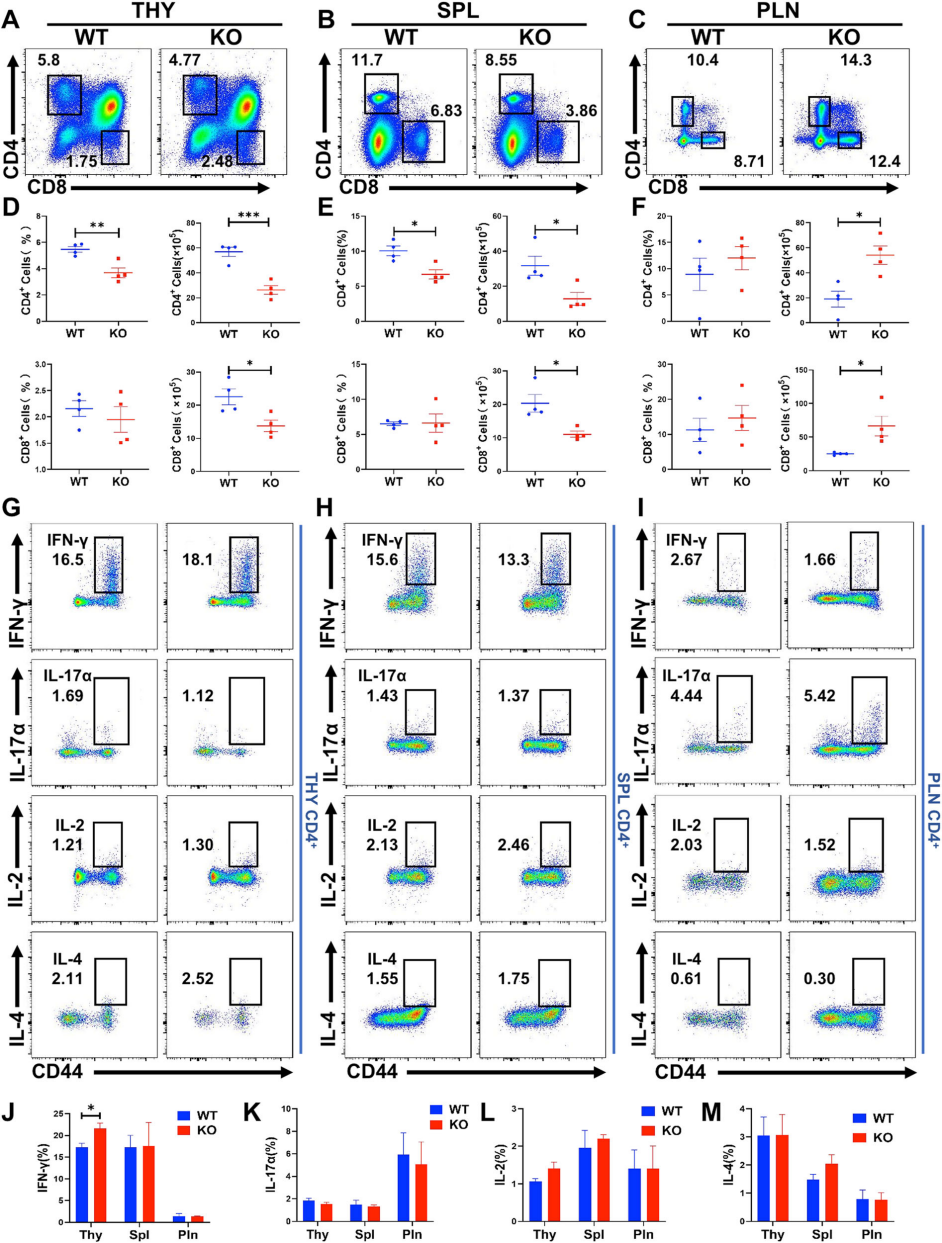
Changes in CD4 and CD8 cells in the thymus, spleen, and lymph nodes of WT and MAPK4 KO mice. **A**–**C** Flow cytometric analysis of the proportions of CD4 and CD8 T cells in the thymus (**A**), spleen (**B**) and lymph nodes (**C**) from WT and MAPK4 KO mice (*n* = 4). **D**–**F** Comparison of the proportions and cell numbers of CD4 and CD8 T cells in the thymus (**D**), spleen (**E**), and lymph nodes (**F**) between WT and MAPK4 KO mice (*n* = 4). **G**–**I** Flow cytometry analysis of IFN-γ, IL-17α, IL-2, and IL-4 levels in stimulated CD4 T cells from the thymus (**G**), spleen (**H**) and lymph nodes (**I**) of WT and MAPK4 KO mice(*n* = 4). **J**–**M** Comparison of IFN-γ (**J**), IL-17α (**K**), IL-2 (**L**), and IL-4 (**M**) levels in CD4 T cells from the thymus, spleen, and lymph nodes between WT and MAPK4 KO mice (*n* = 4). The data are shown as mean ± SEM. *P < 0.05; **P < 0.01; ***P < 0.001.

B cell differentiation and function are closely linked to the state of T cells [28]. To confirm the influence of MAPK4 on T cell subsets, we assessed changes in CD4 and CD8 T cells in the thymus, spleen, and lymph nodes of WT and MAPK4 KO mice using flow cytometry. The percentage of CD4 T cells in the thymus was significantly decreased, while the percentage of CD8 T cells showed a decreasing trend without statistical significance (Fig. 3A, D). Interestingly, the absolute numbers of CD4 and CD8 T cells in the thymus were reduced (Fig. 3A, D). In the spleen, the percentages of both CD4 T cells and the cell numbers of both CD4 and CD8 T cells were reduced, but there was no significant difference in the percentage of CD8 T cells (Fig. 3B, E). In contrast, there was an increase in the absolute numbers of CD4 and CD8 T cells in the peripheral lymph nodes (Fig. 3C, F). Further analysis of CD4 and CD8 T cell subsets involved staining CD62L and CD44 on CD4 and CD8 T cells from various tissues. Notably, there was an increased proportion of effector/memory CD8 T cells in the spleen and lymph nodes of MAPK4 KO mice compared to WT mice. However, no significant differences were observed in other subsets, such as naive and central memory CD4 T cells between WT and MAPK4 KO mice (Fig. S3A–I). Additionally, Treg cells were increased in the lymph nodes of MAPK4 KO mice, while no differences were observed in Treg cells from the spleen and thymus between WT and MAPK4 KO mice (Fig. S3J–O). To further evaluate the cytokine secretion ability of CD4 T cells, we stimulated CD4 T cells in vitro with PMA and ionomycin and detected changes in IL-2, IL-4, IFN-γ, and IL-17 levels. Surprisingly, IFN-γ expressed by CD4 T cells in the thymus of MAPK4 KO mice was higher compared to WT mice, while no significant differences were observed in other cytokines (Fig. 3G–M).

### MAPK4 KO mice exhibit enhanced proximal BCR signaling and B cell activation

**Fig. 4:**
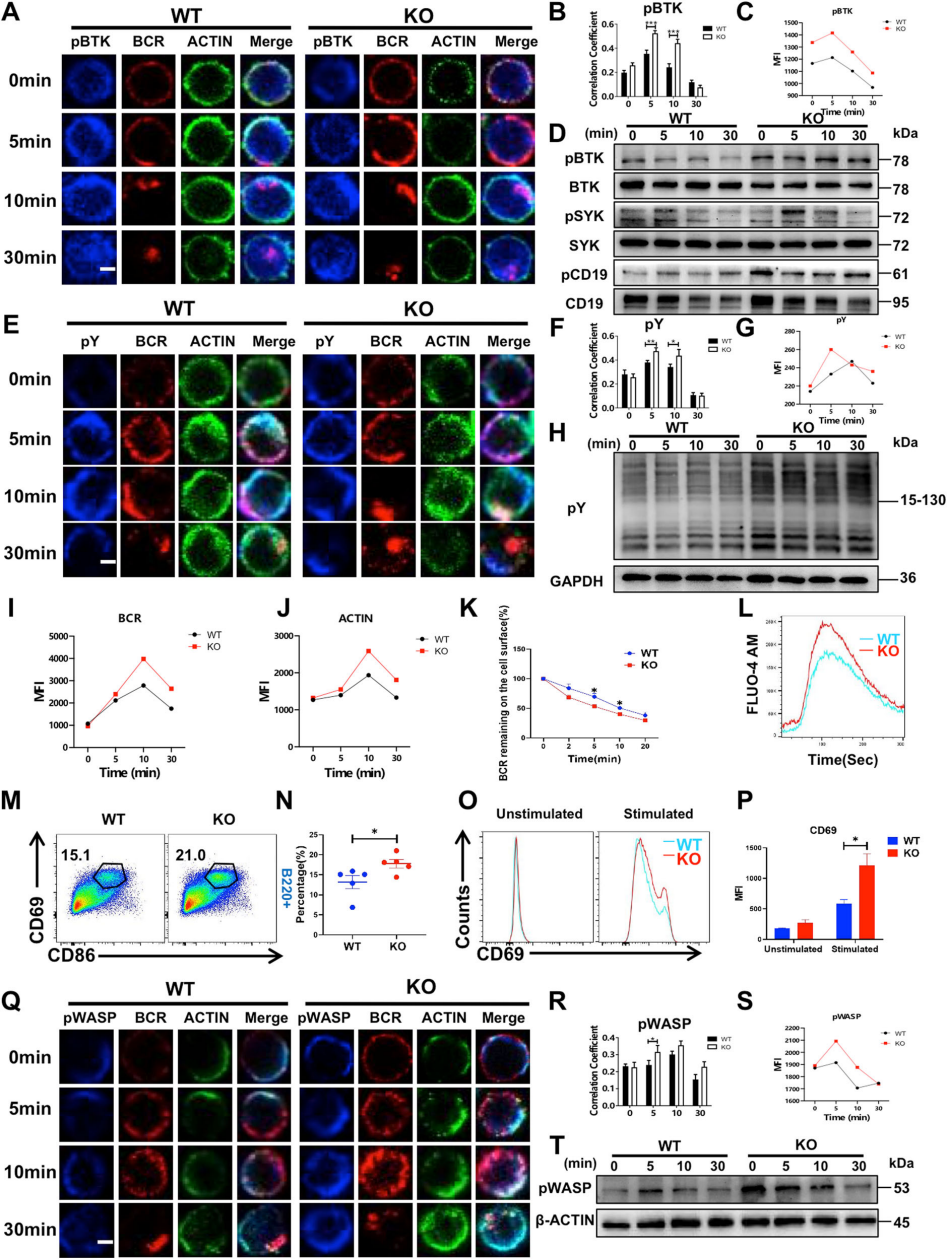
MAPK4 KO mice exhibit enhanced proximal BCR signaling and B cell activation. **A**, **B** Splenic B cells from WT and MAPK4 KO mice were incubated with sAg at different time points, then fixed, permeabilized, and stained for pBTK. Cell images (**A**) were captured using a Leica confocal microscope, and Pearson’s correlation coefficients (**B**) were calculated to evaluate the colocalization of pBTK with the BCR. Scale bar: 2.5 μm. **C** Phospho-flow cytometry analysis of the MFI of pBTK in B cells from WT and MAPK4 KO mice stimulated in vitro with sAg at the indicated time points. **D** Western blot analysis of the pBTK, BTK, pSYK, SYK, pCD19 and CD19 expression levels in B cells from WT and MAPK4 KO mice stimulated in vitro with sAg for 0, 5, 10, and 30 min. **E** Splenic B cells from WT and MAPK4 KO mice were incubated with sAg at different time points, then fixed, permeabilized, and stained for pY. Cell images were captured using a Leica confocal microscope. Scale bar: 2.5 μm. **F** Colocalization coefficients of pY and BCR were compared between WT and MAPK4 KO mice. **G** Phospho-flow cytometry analysis the MFI of pY in B cells from WT and MAPK4 KO mice stimulated in vitro with sAg at the indicated time points. **H** Western blot analysis of pY expression levels in B cells from WT and MAPK4 KO mice stimulated in vitro with sAg for 0, 5, 10, and 30 min. **I**, **J** Flow cytometry analysis the MFI of BCR (**I**) and ACTIN (**J**) in B cells from WT and MAPK4 KO mice stimulated in vitro with sAg at the indicated time points. **K** Flow cytometric analysis of BCR internalization within B cells from WT and MAPK4 KO mice following stimulation with sAg (*n* = 4). **L** Flow cytometry analysis of intracellular calcium levels in B cells from WT and MAPK4 KO mice. **M**, **N** Proportion of CD86^+^CD69^+^B220^+^ B cells (**M**) and comparison (**N**) in B cells from WT and MAPK4 KO mice after 24 h of stimulation with sAg (*n* = 5). **O**, **P** Flow cytometric analysis was performed to compare the expression of CD69 (MFI) in B cells from WT and MAPK4 KO mice after 24 h of stimulation with sAg (*n* = 3). **Q**, **R** Splenic B cells from WT and MAPK4 KO mice were incubated with sAg at different time points, then fixed, permeabilized, and stained for pWASP. Cell images (**Q**) were captured using a Leica confocal microscope, and Pearson’s correlation coefficients (**R**) were calculated to evaluate the colocalization of pWASP with the BCR. Scale bar: 2.5 μm. **S** Phospho-flow cytometry analysis the MFI of pWASP in B cells from WT and MAPK4 KO mice stimulated in vitro with sAg at the indicated time points. **T** Western blot analysis of pWASP expression levels in B cells from WT and MAPK4 KO mice stimulated in vitro with sAg for 0, 5, 10, and 30 min. Representative results from three independent experiments are presented for all of the above. *P < 0.05; **P < 0.01; ***P < 0.001.

The activation of BCR signaling is a key event in the antigen recognition and the subsequent immune response by B cells [29]. To investigate the impact of MAPK4 on BCR signaling, colocalization experiments were first conducted to examine the interaction between the proximal signaling molecule pBTK and BCR in WT and MAPK4 KO mice. After B cells were activated in vitro using sAg, we found a marked increase in the colocalization of pBTK with BCR at the 5 and 10-min marks in MAPK4 KO mice (Fig. 4A, B), suggesting that MAPK4 deficiency enhances the recruitment of pBTK, a key early activation signal molecule, by the BCR. Further analyses using phospho-flow cytometry and Western blot confirmed increased expression levels of pBTK in MAPK4 KO mice post in vitro B cell activation (Fig. 4C, D). Additionally, we examined the expression of other early signal molecules, including pCD19 and pSYK. The results revealed enhanced expression of both pCD19 and pSYK in MAPK4 KO mice post activation of B cells in vitro (Fig. 4D).

To further understand the effects of in vitro B cell activation in MAPK4 KO mice, the colocalization of BCR and pY in WT and MAPK4 KO mice was observed after B cell activation. The results demonstrated a higher colocalization coefficient of pY and BCR in MAPK4 KO mice compared to WT mice (Fig. 4E-F). Further Phospho-flow cytometry and western blot analysis confirmed elevated levels of pY expression in MAPK4 KO mice (Fig. 4G-H). More importantly, flow cytometry analysis showed that the MFIs of BCR and ACTIN were enhanced in MAPK4 KO mice (Fig. 4I-J), which suggests that MAPK4 may inhibit the early signal transduction following B cells activation. Moreover, B cells from MAPK4 KO mice exhibited a significantly increased rate of BCR internalization compared to WT mice upon stimulation with sAg (Fig. 4K). Given that calcium signaling plays a critical role during BCR activation, we further investigated the changes in B cell calcium signaling in WT and MAPK4 KO mice. The results indicated that B cell activation resulted in an enhanced calcium signaling response in MAPK4 KO mice compared to WT mice (Fig. 4L).

To validate the effects of MAPK4 on B cell activation, the expression of CD86 and CD69 on B cells was assessed after 24 h of stimulation with sAg [30]. MAPK4 KO mice exhibited a significant increase in CD86^+^CD69^+^B220^+^ B cells (Fig. 4M, N) and an elevated MFI of CD69 compared to WT mice (Fig. 4O, P), further supporting the observation that MAPK4 KO mice possess stronger BCR signaling and B cell activation.

Moreover, BCR signaling can induce actin polymerization, which is crucial for BCR clustering and efficient signal transduction [31]. An initial flow cytometry examination of actin expression revealed increased actin expression in B cells from MAPK4 KO mice upon activation compared to WT mice (Fig. 4J). Further investigation into the phosphorylation of the actin-regulating protein WASP showed heightened phosphorylation level in MAPK4 KO mice, along with enhanced colocalization with BCRs compared to WT mice (Fig. 4Q–T). These results indicate that B cells from MAPK4 KO mice exhibit enhanced actin polymerization capabilities.

### MAPK4 deficiency activates the PI3K-AKT-mTOR Pathway, enhancing B cell proliferation

**Fig. 5:**
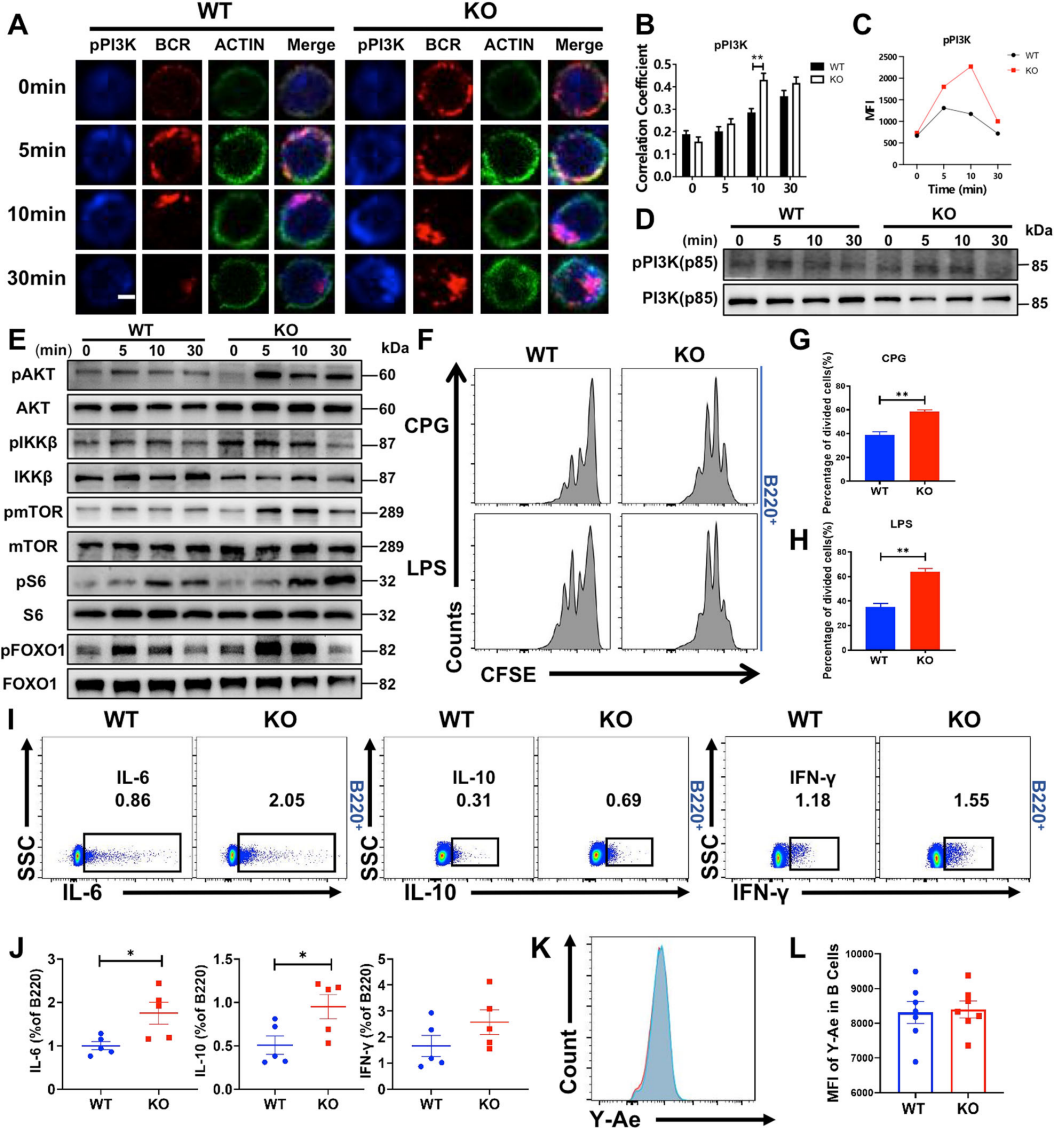
MAPK4 deficiency activates the PI3K-AKT-mTOR pathway, enhancing B cell proliferation. **A**, **B** Splenic B cells from WT and MAPK4 KO mice were incubated with sAg at different time points, then fixed, permeabilized, and stained for pPI3K (p85). Cell images (**A**) were captured using a Leica confocal microscope, and Pearson’s correlation coefficients (**B**) were calculated to evaluate the colocalization of pBTK with the BCR. Scale bar: 2.5 μm. Correlation coefficients were quantified for > 50 cells. **C**, **D** Phospho-flow cytometry (**C**) and western blot (**D**) analysis of pPI3K (p85) expression levels in B cells from WT and MAPK4 KO mice stimulated in vitro with sAg at the indicated time points. **E** Western blot analysis of pAKT, AKT, pIKKβ, IKKβ, pmTOR, mTOR, pS6, S6, pFOXO1 and FOXO1 expression levels in B cells from WT and MAPK4 KO mice stimulated in vitro with sAg for 0, 5, 10, and 30 min. Representative blots from three independent experiments are presented. **F**–**H** Flow cytometric analysis (**F**) and comparison of cell proliferation of naive B cells from WT and MAPK4 KO mice, labeled with CFSE and stimulated with CpG (**G**) or LPS (**H**) in vitro for 72 h (*n* = 3). **I** Flow cytometric analysis of IL-6, IL-10, and IFN-γ expression in B cells from WT and MAPK4 KO mice after stimulation with CPG + anti-CD40, CPG, and PMA + Ionomycin, respectively. **J** Comparison of the proportions of gated populations derived from (**I**) (*n* = 5). **K** Flow cytometric analysis of Eα52–68 peptide presentation by Y-Ae staining in B cells from WT (Cyan) and MAPK4 KO (Red) mice. **L** Comparison of the MFIs of Y-Ae in B cells from WT and MAPK4 KO mice (*n* = 7). *P < 0.05; **P < 0.01; ***P < 0.001.

To explore the distal BCR signaling of pPI3K in WT and KO mice, B cells were analyzed for pPI3K (p85) and BCR colocalization (Fig. 5A). The Pearson correlation coefficient between pPI3K (p85) and BCR colocalization in MAPK4 KO mice was higher than in WT mice (Fig. 5B). In addition, the increased pPI3K (p85) expression was further confirmed by phospho-flow cytometry (Fig. 5C) and Western blot (Fig. 5D). The P13K signal pathway has been proved to play a pivotal role in the physiological response and functions of B cells [32]. Measurement of other downstream signal molecules, including pIKKβ, pAKT, pS6, pmTORC1, pFOXO1, revealed that pIKKβ, pAKT, pS6, pmTORC1, pFOXO1 levels were significantly increased in MAPK4 KO mice compared to WT (Fig. 5E), suggesting that MAPK4 deficiency leads to overactivation of the P13K-AKT-mTOR pathway. The activation of PI3K is closely associated with B cells proliferation. To further investigate the effect of MAPK4 on B-cell proliferation, naive B cells labeled with CFSE were stimulated in vitro with CpG and LPS. After 72 h of stimulation, B cell proliferation was evaluated, and it was found that the proliferation of B cells in MAPK4 KO mice was significantly higher compared to WT mice (Fig. 5F-H). Overall, these findings demonstrate that MAPK4 deficiency activates the PI3K-AKT-mTOR pathway, leading to enhanced B cell proliferation and increased actin polymerization.

Additionally, we addressed the viability of stimulated B cells in vitro. WT and MAPK4 KO B cells were activated with sAg and anti-mouse CD40 for 72 h. Regardless of stimulation, there was no significant difference between the two groups in terms of the percentage of live cells relative to total B cells (Fig. S4A). Furthermore, we found that the proliferation (Ki67) and apoptosis (Annexin V) levels were also unchanged between WT and MAPK4 KO B cells under steady state conditions (Fig. S4A).

Cytokine secretion and antigen presentation by B cells play pivotal roles in the pathogenesis of RA [2]. To investigate these functions, we measured the cytokine secretion capacity of B cells from WT and MAPK4 KO mice. Notably, we found that MAPK4 KO B cells exhibited significantly higher levels of IL-6 and IL-10 than WT B cells, while IFN-γ levels were similar between the two groups (Fig. 5I, J). Additionally, we evaluated the antigen presentation capability of B cells by measuring the levels of Y-Ae [33]. The results of the Ea52-68 peptide presentation, as tested by Y-Ae staining, showed no significant difference between WT and MAPK4 KO B cells (Fig. 5K, L). These findings demonstrate that MAPK4 deficiency specifically increased the cytokine secretion of B cells, but does not alter their antigen presentation ability.

### MAPK4 KO mice exhibit impaired T cell independent humoral immune responses

**Fig. 6:**
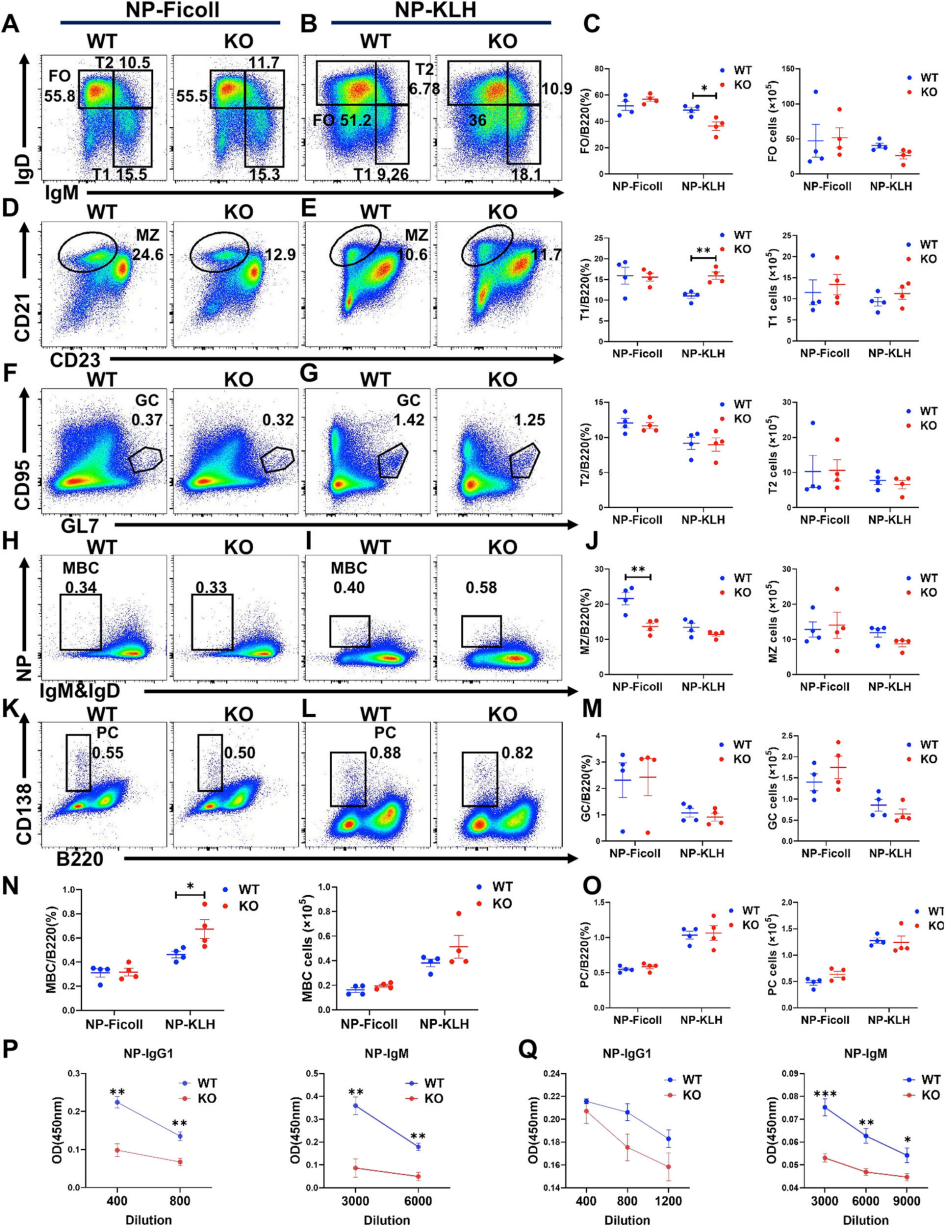
MAPK4 KO mice exhibit impaired TI humoral immune responses. WT and MAPK4 KO mice were immunized with the T cell-independent antigen NP-Ficoll or the T cell-dependent antigen NP-KLH for 14 days, followed by flow cytometry analysis of B cell subpopulations. **A**, **B** Analysis of FO, T1 and T2 cell subpopulations (*n* = 4). **C** Proportions and absolute numbers of FO, T1 and T2 cells (*n* = 4). **D**, **E** Analysis of MZ cell subpopulations (*n* = 4). **F**, **G** Analysis of GC cell subpopulations (*n* = 4). **H**-**I** Analysis of MBC cell subpopulations (*n* = 4). **J** Proportions and absolute numbers of MZ cell (*n* = 4). **K**-**L** Analysis of PC cell subpopulations (*n* = 4). **M** Proportions and absolute numbers of GC cells (*n* = 4). **N** Proportions and absolute numbers of MBC cells (*n* = 4). **O** Proportions and absolute numbers of PC cells (*n* = 4). **P** Specific serum IgM and IgG1 levels were detected by ELISA in WT and MAPK4 KO mice immunized with NP-Ficoll (*n* = 4). **Q** Specific serum IgM and IgG1 levels were detected by ELISA in WT and MAPK4 KO mice immunized with NP-KLH (*n* = 4). *P < 0.05; **P < 0.01; ***P < 0.001.

The activation of BCR induces B cell proliferation, differentiation, and antibody production [34]. Previously, we have shown that MAPK4 is important for MZ B cell differentiation and B cell activation. To further study the physiological effect of MAPK4 in B cell-mediated immune response, WT and MAPK4 KO mice were immunized with TI antigen NP-Ficoll. Mice were analyzed by flow cytometry 14 days post-immunization for B cell subpopulations in the spleen. MAPK4 KO mice had a reduced percentage of MZ B cells compared to WT mice, whereas total B cells, FO, T1, T2, GC, and NP-specific memory B cells, and plasma cells showed no significant difference between these two groups (Fig. 6A–O). However, MAPK4 KO mice have a reduced level of NP-specific IgG1 and IgM in comparison to WT mice (Fig. 6P). Previous studies have shown that PI3K-AKT signaling can downregulate activation-induced cytidine deaminase (AID) expression, leading to impaired class switch recombination (CSR) [35]. To investigate whether this mechanism contributes to the observed reduction in NP-specific IgG1 and IgM production in MAPK4 KO mice, we assessed AID expression in B cells from MAPK4 KO and WT mice following NP-Ficoll immunization. Our results revealed no significant differences in AID expression between the two groups (Fig. S4B). This finding suggests that the impaired humoral immune response in MAPK4 KO mice is not due to altered AID expression but is more likely related to other factors, such as the reduction in MZ B cells, which are essential for T-independent antibody responses.

In the case of T cell-dependent antigen NP-KLH, there were no obvious changes in B cell subpopulations between WT and MAPK4 KO mice at day 14, as determined by flow cytometry analysis, with the exception of a decrease in the percentage of FO B cells and an increase in the percentages of memory B cells and T1 B cells. However, the absolute numbers of these populations showed no significant differences (Fig. 6A–O). MAPK4 KO mice showed lower NP-specific IgM, but no statistically significant changes were observed in IgG1 levels (Fig. 6Q). These data clearly highlight the importance of MAPK4 in regulating humoral immune response. MAPK4 appears to operate differentially in immune response between different antigen classes, with a particularly notably role in T cell-independent immunity mediated via MZ B cells. These findings demonstrate that MAPK4 plays a critical role in regulating humoral immune responses, highlighting its significance in overall immune regulation.

### MAPK4 inhibits the activation of the PI3K signaling pathway in B cells of arthritis mice by activating the IRF4-SHIP1 pathway

**Fig. 7:**
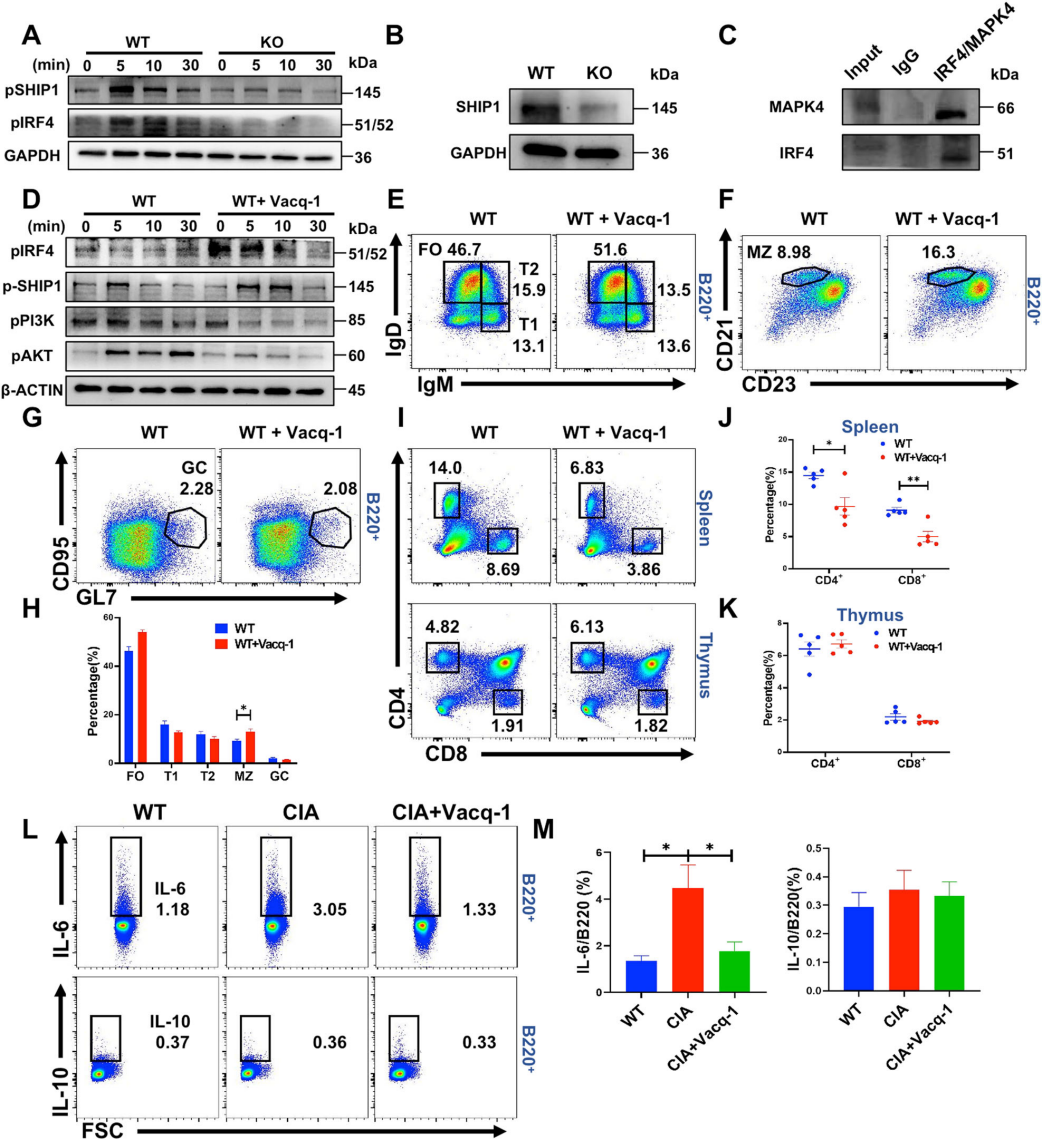
MAPK4 inhibits the activation of the PI3K signaling pathway in B cells of arthritis mice by activating the IRF4-SHIP1 pathway. **A** Western blot analysis of pSHIP1 and pIRF4 expression levels in B cells from WT and MAPK4 KO mice stimulated in vitro with sAg for 0, 5, 10, and 30 min. **B** Western blot analysis of SHIP1 expression in B cells from WT and MAPK4 KO mice. **C** Co-immunoprecipitation assay to analyze the interaction between IRF4 and MAPK4. **D** Western blot analysis of pIRF4, pSHIP1, pPI3K (p85), and pAKT expression levels in WT B cells stimulated in vitro with sAg for 0, 5, 10, and 30 min, with or without Vacquinol-1 treatment. Representative blots from three independent experiments are presented for all of the above. **E**–**H** Flow cytometry (**E**-**G**) and statistical (**H**) analysis of FO(**E**), T1(**E**), T2(**E**), MZ(**F**), and GC (**G**) B cell subpopulations in WT mice treated in vivo with or without Vacquinol-1 every other day for 14 days (*n* = 5). **I**–**K** Flow cytometry (**I**) and statistical analysis (**J**-**K**) of CD4 and CD8 T cells from the spleen and thymus in WT mice treated in vivo with or without Vacquinol-1 every other day for 14 days (*n* = 5). **L**, **M** Flow cytometric analysis of IL-10 and IL-6 expression levels in B cells from WT, CIA, and CIA mice treated in vitro with Vacquinol-1 for 1 h (*n* = 4). *P < 0.05; **P < 0.01; ***P < 0.001.

To further investigate how MAPK4 regulates B cell activation, we first examined the phosphorylation expression of the negative regulatory molecule SHIP1 during B cell activation. The results showed that pSHIP1 levels in B cells from MAPK4 KO mice were significantly lower compared to those from WT mice (Fig. 7A). Given that IRF4 can promote the expression of SHIP1 [36, 37], we further assessed the phosphorylation level of IRF4, which displayed a similar trend to pSHIP1, with reduced expression in MAPK4 KO mice relative to WT mice (Fig. 7A). Western blot analysis further confirmed decreased expression levels of SHIP1 in MAPK4 KO B cells compared to their WT counterparts (Fig. 7B). To determine whether MAPK4 directly regulates the phosphorylation of IRF4, we conducted co-immunoprecipitation assays, which provided compelling evidence of an interaction between MAPK4 and IRF4 (Fig. 7C). This finding suggests a direct regulatory role of MAPK4 in IRF4 phosphorylation.

To further corroborate that MAPK4 inhibits B cell activation via the IRF4-SHIP1 signaling pathway, we employed a MAPK4-specific activator, Vacquinol-1, to stimulate B cells from WT mice in vitro. Remarkably, Vacquinol-1 treatment led to increased expression of phosphorylated IRF4 and SHIP1, while concurrently decreasing the expression levels of phosphorylated PI3K and AKT (Fig. 7D), indicating a suppressive effect of MAPK4 on B cell activation. To investigate the effect of the MAPK4 agonist Vacquinol-1 on B cell differentiation in vivo, mice were administered intraperitoneal injections of Vacquinol-1 to mice every other day for 14 days. The results showed a significant increase in MZ B cell differentiation, with no significant alterations observed in other B cell subsets such as FO, T1, T2, and GC B cells (Fig. 7E–H). Intriguingly, Vacquinol-1 stimulation resulted in a reduction in the proportion of CD4 and CD8 T cells (Fig. 7I–K) but showed no impact on IL-4, IL-17 and IFN-γ secretion (Fig. S4C–F) of CD4 and CD8 T cells from the spleen compared to WT mice.

Collectively, these findings suggest that MAPK4 plays a negative regulatory role in B cell activation and MZ B cell differentiation through the promotion of the IRF4-SHIP1 signaling pathway. Furthermore, in a CIA mouse model, treatment of B cells with the MAPK4 agonist Vacquinol-1 in vitro significantly reduced IL-6 secretion, while IL-10 levels remained unchanged (Fig. 7L, M), indicating that Vacquinol-1 effectively suppresses IL-6 production by CIA mice B cells.

## Discussion

In this study, we thoroughly investigated the crucial role of MAPK4 in early B cell activation and function, particularly in the context of RA. Abnormal early activation of B cells was observed in RA patients and CIA mice, accompanied by reduced expression of MAPK4 in B cells. We first confirmed that MAPK4 promotes MZ B cell differentiation through a cell-intrinsic pathway. Furthermore, we demonstrated that MAPK4 is involved in suppressing early B cell activation. The knockout of MAPK4 led to increased expression of both proximal and distal activation molecules in B cells, which is critical for the immune response to T cell-independent (TI) antigens. Mechanistically, we found that MAPK4 can suppress early B cell activation by activating the IRF4-SHIP1 pathway. Importantly, the MAPK4 specific agonist Vacquinol-1 was also effective in promoting MZ B cell differentiation in WT mice and in reducing IL-6 production by B cells in CIA mice. In summary, the results of this study suggest that MAPK4 plays various roles in the regulation of B cell function, with potential implications for the development of therapeutic strategies targeting RA and related autoimmune diseases.

Our study contributes novel insights into the role of MAPK4 in B cell regulation and its implications for autoimmune diseases such as RA. Notably, our findings regarding the enhanced B cell proliferation and actin polymerization due to MAPK4 deficiency align with emerging research on the MAPK4 pathway’s broader impact on cell biology. For instance, a study by Zhang et al. on breast cancer cells identified the MAPK4 axis as crucial for cell proliferation, and migration, suggesting a conserved role of MAPK4 in regulating cell dynamics across different cell types, including B cells [38]. Furthermore, our observation that MAPK4 KO mice exhibit impaired humoral immune responses extends the understanding of MAPK4’s function beyond its previously reported roles in tumor biology and highlights its significance in immune regulation. After NP-Ficoll immunization, a reduced level of NP-specific IgG_1_ and IgM were observed in MAPK4 KO mice. The reduction of MZ B cells, which are responsible for the early production of IgM and IgG_1_ triggered by T-independent antigens, may explain this observed decrease. Notably, although AID expression remained unchanged, the reduced number of MZ B cells might impair the potential for AID-associated class-switch recombination to IgG_1_ switch recombinant genes. These results suggest that the MAPK4 pathway is critical for regulating both the development of MZ B cells and their functional responses, particularly to TI antigens.

Our research also sheds light on the negative regulatory role of MAPK4 in the activation of the PI3K signaling pathway in B cells through the IRF4-SHIP1 pathway. While earlier research has often highlighted the positive regulatory mechanisms of MAPK4 in various signaling pathways [17], our results suggest a complex, context-dependent regulation of immune responses by MAPK4, particularly in the setting of autoimmune diseases like RA. This is indicative of the complexity and importance of further research into the molecular mechanism of MAPK4 and its potential as a therapeutic target.

Over time, the RA-targeted therapeutic landscape has shifted attention toward the inhibition of pro-inflammatory pathways and cellular processes central to the disease’s pathogenesis. Common targets include tumor necrosis factor-alpha (TNF-α) [39], interleukin-6 (IL-6) receptors [40], and Janus kinases (JAKs) [41], which have led to the development of biologics and small molecule inhibitors that have significantly transformed RA management. Despite these advances, improved therapeutic measures that are less toxic, more effective, and capable to modifying disease progression are still being sought, since patients often show incomplete response or develop side effects. MAPK4 plays a dual role in regulating B-cell activation and proliferation while influencing the PI3K-AKT-mTOR pathway, making it a promising therapeutic target for RA. Developing MAPK4 agonists may offer a strategy to modulate B cell function and effector activity, preventing widespread systemic immunity without causing generalized immune suppression. This approach has the potential to modify disease progression and reduce the toxicity associated with current RA treatments. However, these findings will need to be put into practice by large translational investigations. Future research will be needed to determine the precise molecular mechanisms through which MAPK4 agonists can shift immune responses in RA, including specific effects on B cell subpopulations, signaling pathways, and overall disease course and symptomatology. Moreover, safety profile with regard to MAPK4 targeting in terms of off-target effects and long-term consequences due to pathway modulation, in order to design a therapeutic agent for RA, which is safe for use with these RA patients.

Although the study provides key insights into the role of MAPK4 in B cells and its possible application for the treatment of RA, it also has its shortcomings. Above all, the major limitation lies in the use of constitutive global MAPK4 knockouts, although informative, in relation to the dissecting of tissue- and cell-specific actions brought about by MAPK4 deficiency. A global knockout may mask compensatory mechanisms or side effects resulting from the lack of MAPK4 in other cell types and tissues, which may then bias the interpretation of its role in B cells and, more generally, immunity. To overcome these limitations and further dissect the role of MAPK4 in RA, future research has to be conducted using conditional knockout mice that precisely delete MAPK4 in B cells or in other specific cell populations of interest. This will further help us define the cell-autonomous role of MAPK4 in general and in the immune system, and its contribution to the dysregulation in the pathogenesis of RA. In turn, conditional knockouts of B cells will provide insight into cell type-specific functions of MAPK4, possible modes of regulation of MAPK4 activity, and possibly even direct targets of MAPK4 that mediate these functions within B cells. Again, it is through conditional knockouts that the granularity of MAPK4 liaising with other signaling molecules and pathways within B cells can be established. This will further consolidate MAPK4 as a true therapeutic target and an understanding of B cell biology and its perturbation in autoimmune diseases.

In summary, our study elucidates the crucial role of MAPK4 in B cell function and its implications for RA. We demonstrate that reduced expression of MAPK4 significantly affects B cell differentiation, humoral immune responses, and key signaling pathways, positioning MAPK4 as a novel potential therapeutic target for RA. This work lays the groundwork for the development of MAPK4-targeted treatments, offering a promising direction for improving RA management and advancing our understanding of autoimmune disease mechanisms.

## Supplementary information

Supplementary Figures

Supplementary Table S1

Original Data File

## Data Availability

All data generated or analysed during this study are included in this published article and its supplementary information files.

## References

[1] Ebel AV, O’Dell JR. Clinical features, diagnosis, and treatment of rheumatoid arthritis. Phys Assist Clin. 2021;6:41–60.

[2] Wu F, Gao J, Kang J, Wang X, Niu Q, Liu J, et al. B cells in rheumatoid arthritis: pathogenic mechanisms and treatment prospects. Front Immunol. 2021;12:750753. 34650569 10.3389/fimmu.2021.750753 PMC8505880

[3] Kristyanto H, Blomberg NJ, Slot LM, van der Voort EI, Kerkman PF, Bakker A, et al. Persistently activated, proliferative memory autoreactive B cells promote inflammation in rheumatoid arthritis. Sci Transl Med. 2020;12:eaaz5327. 33208502 10.1126/scitranslmed.aaz5327 PMC7615909

[4] Lee DS, Rojas OL, Gommerman JL. B cell depletion therapies in autoimmune disease: advances and mechanistic insights. Nat Rev Drug Discov. 2021;20:179–99. 33324003 10.1038/s41573-020-00092-2 PMC7737718

[5] Richez C, Truchetet M-E, Schaeverbeke T, Bannwarth B. Atacicept as an investigated therapy for rheumatoid arthritis. Expert Opin Inv Drug. 2014;23:1285–94. 10.1517/13543784.2014.943835 25078871

[6] Tanaka S, Baba YB. cell receptor signaling. In: Wang, JY (ed) Cells in immunity and tolerance. advances in experimental medicine and biology, Springer, pp 1254 10.1007/978-981-15-3532-1_2 32323266

[7] Tolar P. Cytoskeletal control of B cell responses to antigens. Nat Rev Immunol. 2017;17:621–34. 28690317 10.1038/nri.2017.67

[8] Browne CD. Molecular mechanisms of B cell tolerance, proliferation and motility[D]. La Jolla, CA United States: University of California, San Diego; 2010.

[9] Rawlings DJ, Metzler G, Wray-Dutra M, Jackson SW. Altered B cell signalling in autoimmunity. Nat Rev Immunol. 2017;17:421–36. 28393923 10.1038/nri.2017.24 PMC5523822

[10] Fedele AL, Tolusso B, Gremese E, Bosello SL, Carbonella A, Canestri S, et al. Memory B cell subsets and plasmablasts are lower in early than in long-standing rheumatoid arthritis. BMC Immunol. 2014;15:1–9. 25187226 10.1186/s12865-014-0028-1 PMC4168163

[11] H Khorsheed S, F Mustafa I, A Naji N. Study the activity of tryptase and beta 2-microglobulin levels in the sera of patients with chronic renal failure and rheumatoid arthritis. Kirkuk J Sci. 2016;11:138–54.

[12] De Rooy DP, Willemze A, Mertens B, Huizinga TW, Van der Helm-van Mil AH. Can anti-cyclic citrullinated peptide antibody-negative RA be subdivided into clinical subphenotypes? Arthritis Res Ther. 2011;13:1–7. 10.1186/ar3505 PMC3308115 22032620

[13] Engel P, Gómez-Puerta JA, Ramos-Casals M, Lozano F, Bosch X. Therapeutic targeting of B cells for rheumatic autoimmune diseases. Pharmacol Rev. 2011;63:127–56. 21245206 10.1124/pr.109.002006

[14] Neys SF, Heutz JW, van Hulst JA, Vink M, Bergen IM, de Jong PH, et al. Aberrant B cell receptor signaling in circulating naïve and IgA+ memory B cells from newly-diagnosed autoantibody-positive rheumatoid arthritis patients. J Autoimmun. 2024;143:103168. 38350168 10.1016/j.jaut.2024.103168

[15] Ding Q, Hu W, Wang R, Yang Q, Zhu M, Li M, et al. Signaling pathways in rheumatoid arthritis: implications for targeted therapy. Signal Transduct Tar. 2023;8:68. 10.1038/s41392-023-01331-9 PMC9935929 36797236

[16] Ronkina N, Gaestel M. MAPK-activated protein kinases: servant or partner? Annu Rev Biochem. 2022;91:505–40. 35303787 10.1146/annurev-biochem-081720-114505

[17] Yan Y, Dai T, Guo M, Zhao X, Chen C, Zhou Y, et al. A review of non-classical MAPK family member, MAPK4: a pivotal player in cancer development and therapeutic intervention. Int J Biol Macromol. 2024;271:132686. 38801852 10.1016/j.ijbiomac.2024.132686

[18] Zhao J, Chu F, Xu H, Guo M, Shan S, Zheng W, et al. C/EBPα/miR‐7 controls CD4+ T‐cell activation and function and orchestrates experimental autoimmune hepatitis in mice. Hepatology. 2021;74:379–96. 33125780 10.1002/hep.31607

[19] Lu H, Yang Y, Ou S, Qi Y, Li G, He H, et al. The silencing of miR-199a-5p protects the articular cartilage through MAPK4 in osteoarthritis. Ann Transl Med. 2022;10:601. 35722355 10.21037/atm-22-2057 PMC9201181

[20] Mao L, Zhou Y, Chen L, Hu L, Liu S, Zheng W, et al. Identification of atypical mitogen-activated protein kinase MAPK4 as a novel regulator in acute lung injury. Cell Biosci. 2020;10:1–17. 33088477 10.1186/s13578-020-00484-2 PMC7570399

[21] Du Z, Yang D, Zhang Y, Xuan X, Li H, Hu L, et al. AKT2 deficiency impairs formation of the BCR signalosome. Cell Commun Signal. 2020;18:56. 32252758 10.1186/s12964-020-00534-9 PMC7133013

[22] Du Z, Chen A, Huang L, Dai X, Chen Q, Yang D, et al. STAT3 couples with 14-3-3σ to regulate BCR signaling, B-cell differentiation, and IgE production. J Allergy Clin Immun. 2021;147:1907–23.e6. 33045280 10.1016/j.jaci.2020.09.033

[23] Caplazi P, Baca M, Barck K, Carano R, DeVoss J, Lee W, et al. Mouse models of rheumatoid arthritis. Vet Pathol. 2015;52:819–26. 26063174 10.1177/0300985815588612

[24] Jing Y, Dai X, Yang L, Kang D, Jiang P, Li N, et al. STING couples with PI3K to regulate actin reorganization during BCR activation. Sci Adv. 2020;6:eaax9455. 32494627 10.1126/sciadv.aax9455 PMC7176427

[25] Zhang P, Ruan C, Yang G, Guan Y, Zhu Y, Li Q, et al. PGRN inhibits early B-cell activation and IgE production through the IFITM3-STAT1 signaling pathway in asthma. Adv Sci. 2024;11:e2403939. 10.1002/advs.202403939 PMC11615816 39412083

[26] Thorarinsdottir K, Camponeschi A, Jonsson C, Granhagen Önnheim K, Nilsson J, Forslind K, et al. CD21−/low B cells associate with joint damage in rheumatoid arthritis patients. Scand J Immunol. 2019;90:e12792. 31141193 10.1111/sji.12792

[27] Rincón-Arévalo H, Rojas M, Vanegas-García A, Muñoz-Vahos C, Orejuela-Erazo J, Vásquez G, et al. Atypical phenotype and response of B cells in patients with seropositive rheumatoid arthritis. Clin Exp Immunol. 2021;204:221–38. 33459349 10.1111/cei.13576 PMC8062998

[28] Egawa T, Bhattacharya D. Regulation of metabolic supply and demand during B cell activation and subsequent differentiation. Curr Opin Immunol. 2019;57:8–14. 30339937 10.1016/j.coi.2018.10.003 PMC6467717

[29] Chen Z, Wang JH. How the signaling crosstalk of B cell receptor (BCR) and co-receptors regulates antibody class switch recombination: a new perspective of checkpoints of BCR signaling. Front Immunol. 2021;12:663443. 33841447 10.3389/fimmu.2021.663443 PMC8027318

[30] Barrio L, Román-García S, Díaz-Mora E, Risco A, Jiménez-Saiz R, Carrasco YR, et al. B cell development and T-dependent antibody response are regulated by p38γ and p38δ. Front Cell Dev Biol. 2020;8:189. 32266269 10.3389/fcell.2020.00189 PMC7105866

[31] Li N, Jiang P, Chen A, Luo X, Jing Y, Yang L, et al. CX3CR1 positively regulates BCR signaling coupled with cell metabolism via negatively controlling actin remodeling. Cell Mol Life Sci. 2020;77:4379–95. 32016488 10.1007/s00018-019-03416-7 PMC11105092

[32] Jellusova J, Rickert RC. The PI3K pathway in B cell metabolism. Crit Rev Biochem Mol. 2016;51:359–78. 10.1080/10409238.2016.1215288 PMC5139348 27494162

[33] Jing Z, McCarron MJ, Dustin ML, Fooksman DR. Germinal center expansion but not plasmablast differentiation is proportional to peptide-MHCII density via CD40-CD40L signaling strength. Cell Rep. 2022;39:110763. 35508132 10.1016/j.celrep.2022.110763 PMC9178878

[34] Liu W, Tolar P, Song W, Kim TJ. Editorial: BCR signaling and B cell activation. Front Immunol. 2020;11:45. 32063903 10.3389/fimmu.2020.00045 PMC6999073

[35] Chen Z, Getahun A, Chen X, Dollin Y, Cambier JC, Wang JH. Imbalanced PTEN and PI3K signaling impairs class switch recombination. J Immunol. 2015;195:5461–71. 26500350 10.4049/jimmunol.1501375 PMC4655169

[36] Falasca M, Logan S, Lehto V, Baccante G, Lemmon M, Schlessinger J. Activation of phospholipase Cγ by PI 3‐kinase‐induced PH domain‐mediated membrane targeting. EMBO J. 1998;17:414–22. 9430633 10.1093/emboj/17.2.414 PMC1170392

[37] Wen Y, Jing Y, Yang L, Kang D, Jiang P, Li N, et al. The regulators of BCR signaling during B cell activation. Blood Sci. 2019;1:119–29. 35402811 10.1097/BS9.0000000000000026 PMC8975005

[38] Wang W, Han D, Cai Q, Shen T, Dong B, Lewis MT, et al. MAPK4 promotes triple negative breast cancer growth and reduces tumor sensitivity to PI3K blockade. Nat Commun. 2022;13:1–14. 35017531 10.1038/s41467-021-27921-1 PMC8752662

[39] Du H, Wang Y, Zeng Y, Huang X, Liu D, Ye L, et al. Tanshinone IIA suppresses proliferation and inflammatory cytokine production of synovial fibroblasts from rheumatoid arthritis patients induced by TNF-α and attenuates the inflammatory response in AIA mice. Front Pharmacol. 2020;11:568. 32499694 10.3389/fphar.2020.00568 PMC7243269

[40] Nouri B, Nair N, Barton A. Predicting treatment response to IL6R blockers in rheumatoid arthritis. Rheumatology. 2020;59:3603–10. 32864695 10.1093/rheumatology/keaa529 PMC7733712

[41] Tanaka Y, Luo Y, O’Shea JJ, Nakayamada S. Janus kinase-targeting therapies in rheumatology: a mechanisms-based approach. Nat Rev Rheumatol. 2022;18:133–45. 34987201 10.1038/s41584-021-00726-8 PMC8730299

