# Supplementary material for: MAPK4 inhibits the early aberrant activation of B cells in rheumatoid arthritis by promoting the IRF4-SHIP1 signaling pathway
Source: Cell Death Dis. 2025 Jan 26;16(1):43. doi: 10.1038/s41419-025-07352-2 (PMC11763251; doi:10.1038/s41419-025-07352-2)
Supplement: Supplementary file 1 — Supplementary Figures [file 41419_2025_7352_MOESM1_ESM.pdf]

Supplementary Figure Legends:

Figure S1

A. Western blot analysis of MAPK4 expression in splenic B cells from WT and MAPK4 KO mice. Representative blots from three independent experiments are presented. **B-D.** Flow cytometric analysis of the percentages and absolute numbers of bone marrow B cell subpopulations in WT and MAPK4 KO bone marrow chimera mice, including pre-pro-B cells (A), pro-B cells (B), early pre-B cells (C), late pre-B cells (D), immature B cells (E), and recirculating B (F) cells (n = 3). **E-F.** Flow cytometric analysis of proportions and absolute numbers splenic B cells in WT and MAPK4 KO bone marrow chimera mice (n = 3). **G-P.** Flow cytometric analysis of splenic B cell subpopulations in WT and MAPK4 mice. The proportions and absolute numbers of B1a (**G & H**), B1b (**G & H**), FO (**I & J**), T1(**I & K**), T2 (**I & L**), MZ (**M & N**), and GC (**O & P**) cells were compared between WT and MAPK4 KO bone marrow chimera mice (n = 3). All data are presented as mean ± SEM. *: $P < 0.05$.

Figure S2

A-D. Flow cytometric evaluation of Annexin V (**A & C**) and Ki67 (**B & D**) positive rates in B cells and their respective subsets in both WT and MAKP4 KO mice (n = 4). Data are presented as mean ± SEM.

Figure S3

A-I. Flow cytometric analysis of CD4 and CD8 T cell subsets in the spleen (**left**), thymus (**middle**) and lymph nodes (**right**) from WT and MAPK4 KO mice (n = 6), including spleen CD4 Tnaive, Tem and Tcm cells (**A & D**), spleen CD8 Tnaive, Tem and Tcm cells (**A & G**), thymus CD4 Tnaive, Tem and Tcm cells (**B & E**), thymus CD8 Tnaive, Tem and Tcm cells (**B & H**), peripheral lymph nodes CD4 Tnaive, Tem and Tcm cells (**C & F**), and peripheral lymph nodes CD8 Tnaive, Tem and Tcm cells (**C & I**). **J-O**. Flow cytometric analysis of proportions of $CD4^{+}FOXP3^{+}$ in the spleen

(**J & M**), thymus (**K & N**) and lymph nodes (**L & O**) from WT and MAPK4 KO mice (n = 4). *: $P < 0.05$; **: $P < 0.01$; ***: $P < 0.001$.

Figure S4

A. Flow cytometric analysis of Annexin V, Ki67, and zombie NIR live cells among B cells from WT and MAPK4 KO mice, with or without stimulation by sAg and anti-mouse CD40 for 72-hour (n = 4). **B**. Western blot analysis of AID expression in B cells isolated from WT and MAPK4 KO mice 14 days after immunization with NP-Ficoll. Representative results from three independent experiments are presented. **C-F.** Flow cytometric analysis of IL-4, IL-17 and IFN-γ secreted by CD4 (**C & E**) and CD8 (**D & F**) cells from WT and Vacquinol-1 simulated WT mice *in vivo* for 14 days (n = 5).

Figure S1

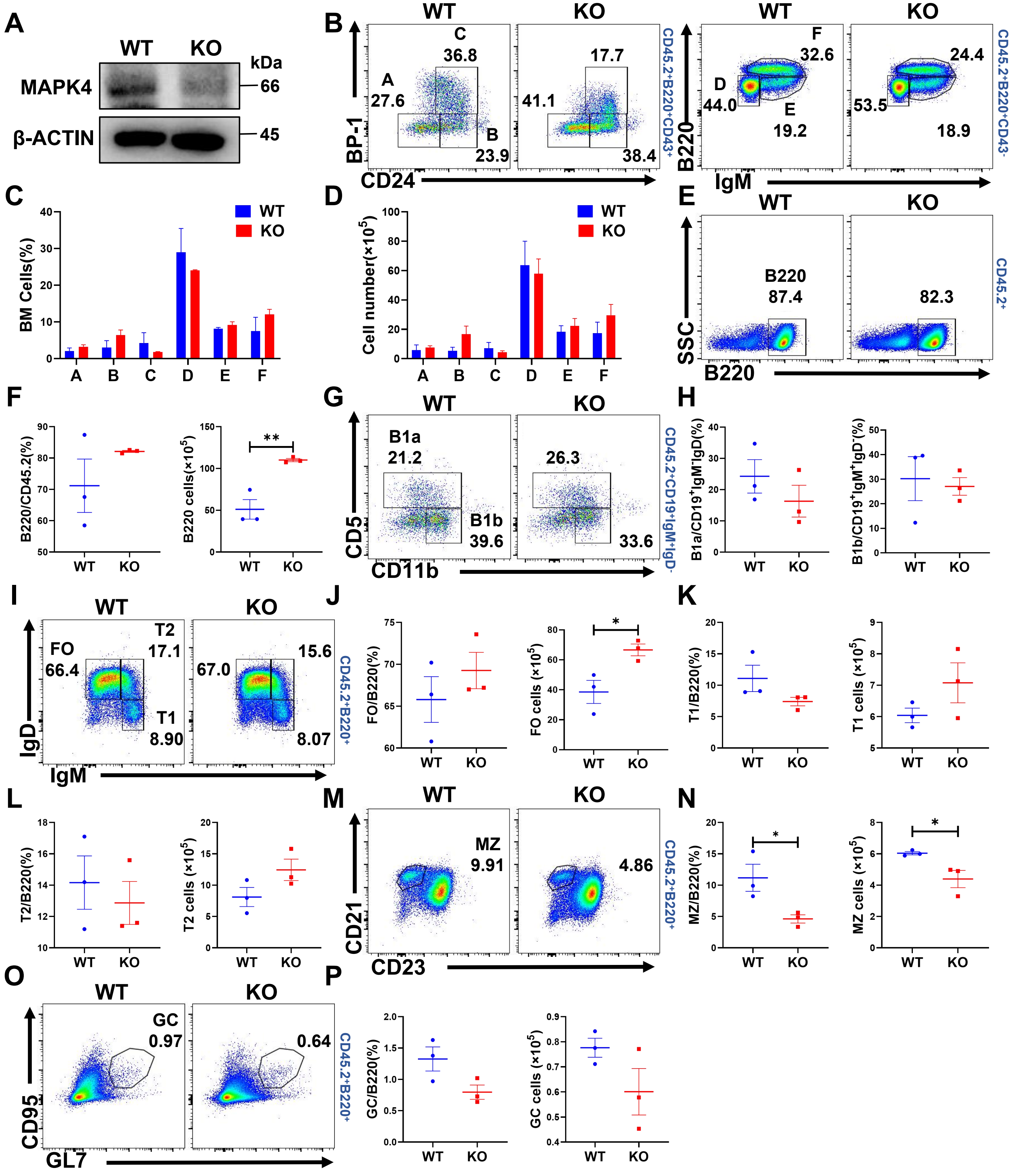

A
WT
KO
kDa
MAPK4
66
β-ACTIN
45
B
WT
KO
BP-1
CD24
A 27.6
C 36.8
B 23.9
41.1
17.7
38.4
CD45.2+B220+CD43+
B220
IgM
D 44.0
F 32.6
E 19.2
53.5
24.4
18.9
CD45.2+B220+CD43-
C
BM Cells(%)
D
Cell number(×10^5)
E
SSC
B220
B220 87.4
82.3
CD45.2+
F
B220/CD45.2(%)
B220 cells(×10^5)
G
CD5
CD11b
B1a 21.2
B1b 39.6
26.3
33.6
CD45.2+CD19+IgM+IgD-
H
B1a/CD19+IgM-IgD(%)
B1b/CD19+IgM+IgD-(%)
I
IgD
IgM
FO 66.4
T2 17.1
T1 8.90
67.0
15.6
8.07
CD45.2+B220+
J
FO/B220(%)
FO cells (×10^5)
K
T1/B220(%)
T1 cells (×10^5)
L
T2/B220(%)
T2 cells (×10^5)
M
CD21
CD23
MZ 9.91
4.86
N
MZ/B220(%)
MZ cells (×10^5)
O
CD95
GL7
GC 0.97
0.64
P
GC/B220(%)
GC cells (×10^5)

Figure S2

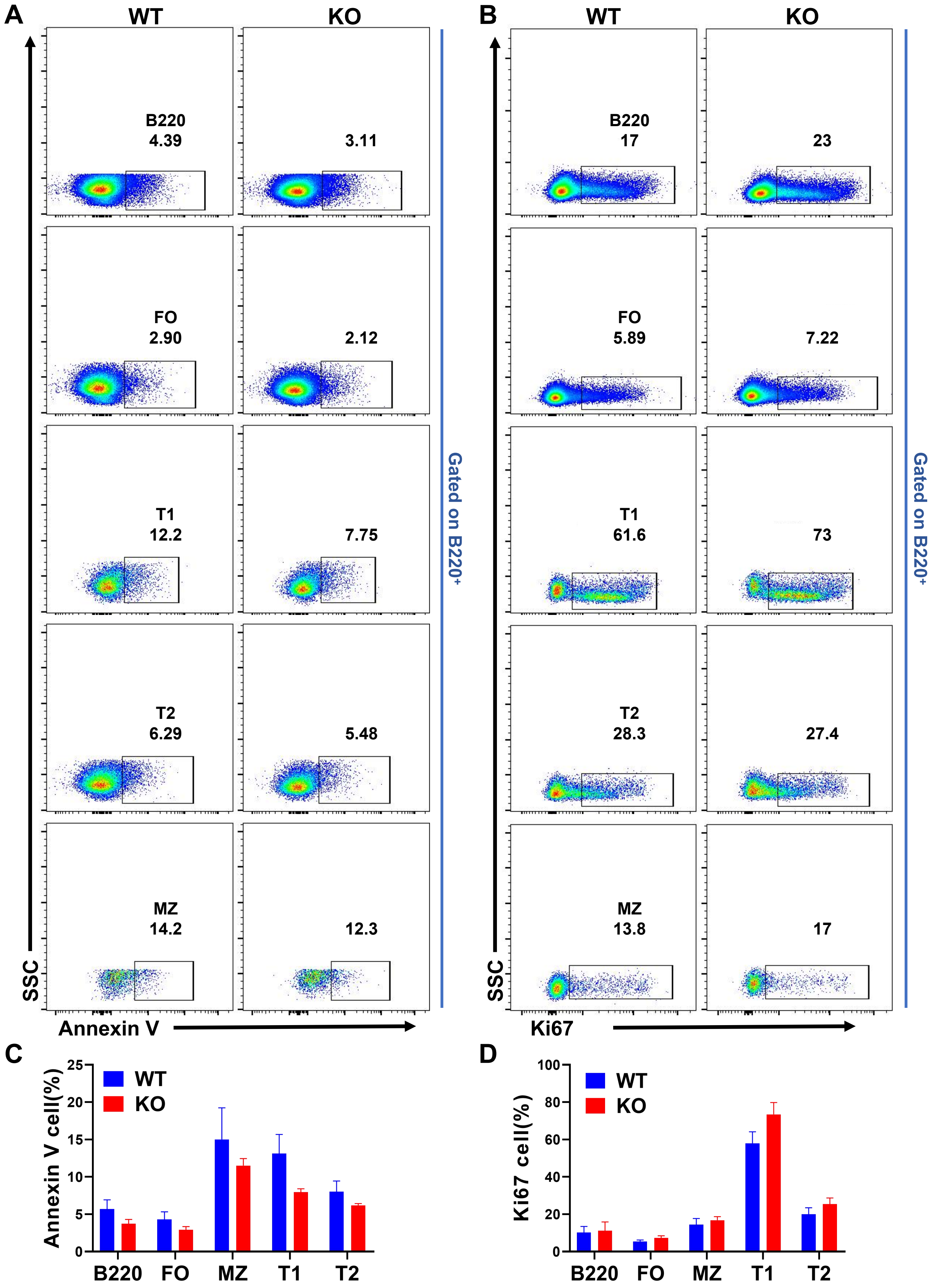
A
WT
KO
B220
4.39
3.11
FO
2.90
2.12
T1
12.2
7.75
T2
6.29
5.48
MZ
14.2
12.3
SSC
Annexin V
Gated on B220+
B
WT
KO
B220
17
23
FO
5.89
7.22
T1
61.6
73
T2
28.3
27.4
MZ
13.8
17
SSC
Ki67
Gated on B220+
C
WT
KO
Annexin V cell(%)
0
5
10
15
20
25
B220
FO
MZ
T1
T2
D
WT
KO
Ki67 cell(%)
0
20
40
60
80
100
B220
FO
MZ
T1
T2

Figure S3

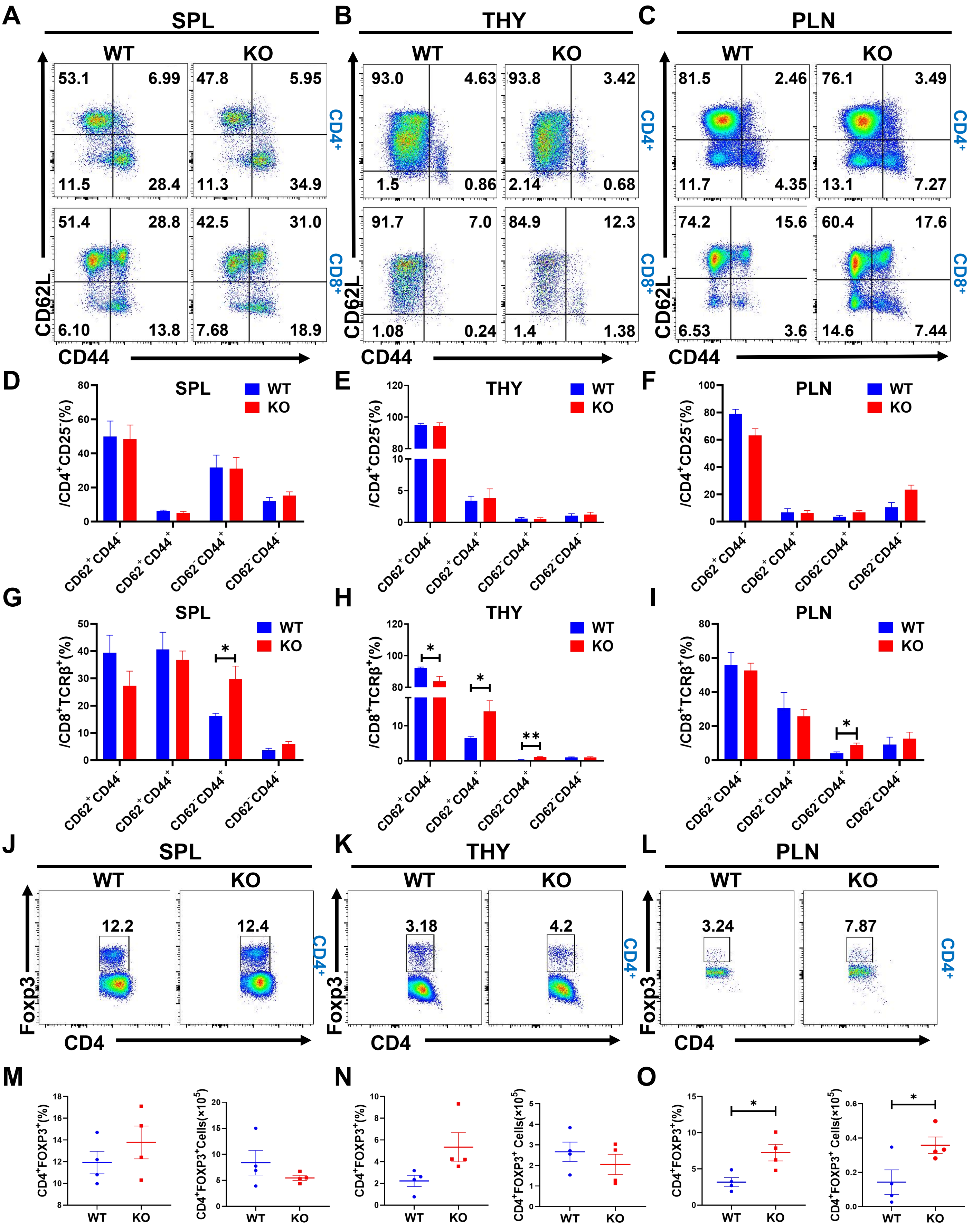

A SPL
B THY
C PLN
WT
KO
CD4+
CD8+
CD62L
CD44
D SPL
E THY
F PLN
/CD4+CD25-(%)
CD62+CD44-
CD62+CD44+
CD62-CD44+
CD62-CD44-
G SPL
H THY
I PLN
/CD8+TCRβ+(%)
J SPL
K THY
L PLN
Foxp3
CD4
M
N
O
CD4+FOXP3+(%)
CD4+FOXP3+Cells(×10^5)

Figure S4

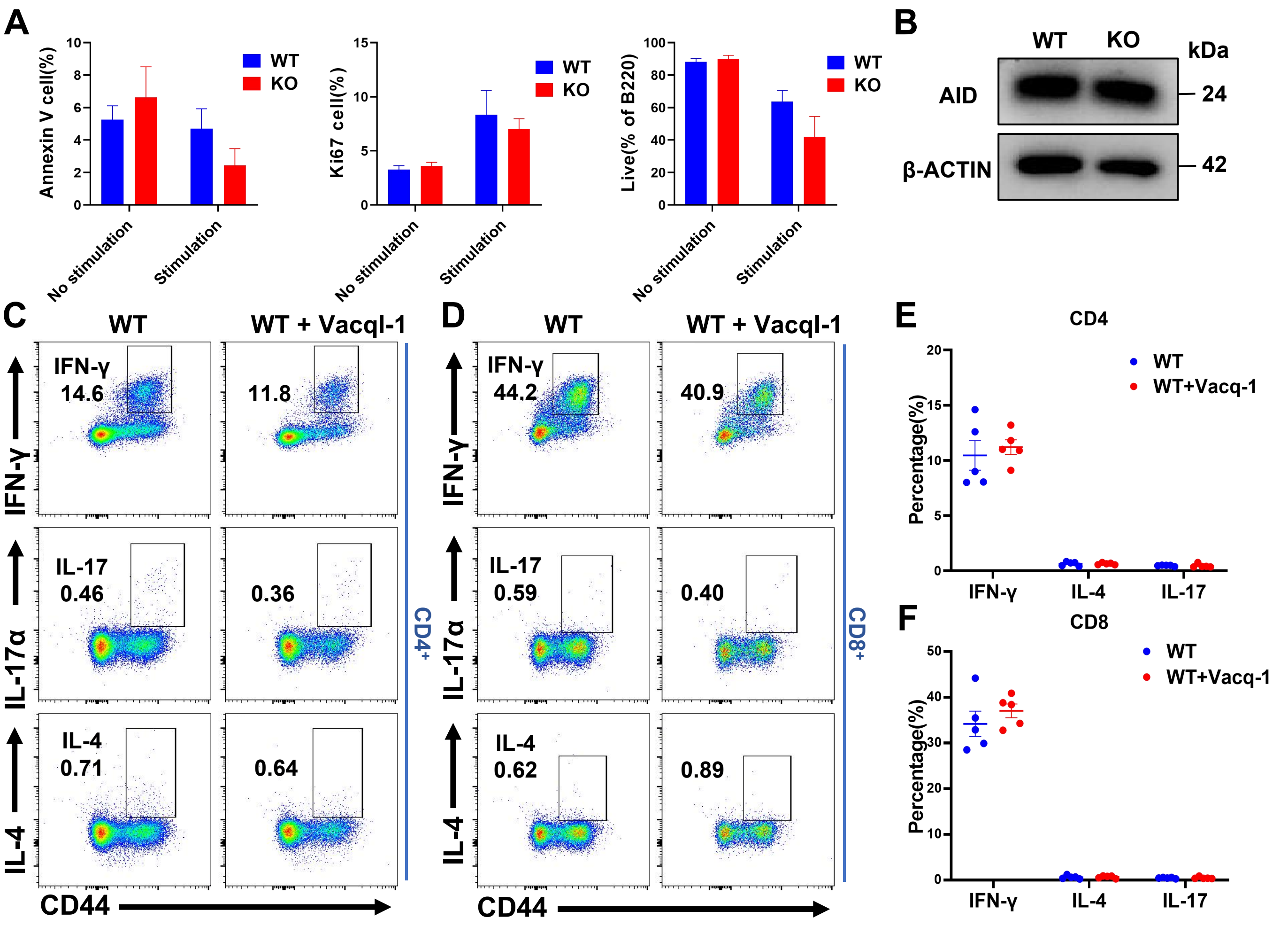
A
Annexin V cell(%)
WT
KO
No stimulation
Stimulation
Ki67 cell(%)
Live(% of B220)
B
WT
KO
kDa
AID
24
β-ACTIN
42
C
WT
WT + Vacql-1
IFN-γ
14.6
11.8
IL-17
0.46
0.36
IL-4
0.71
0.64
IL-17α
CD44
CD4+
D
44.2
40.9
0.59
0.40
0.62
0.89
CD8+
E
CD4
WT+Vacq-1
Percentage(%)
F
CD8
