# Supplementary material for: MAPK4 inhibits the early aberrant activation of B cells in rheumatoid arthritis by promoting the IRF4-SHIP1 signaling pathway
Source: Cell Death Dis. 2025 Jan 26;16(1):43. doi: 10.1038/s41419-025-07352-2 (PMC11763251; doi:10.1038/s41419-025-07352-2)

Source Fig.1G

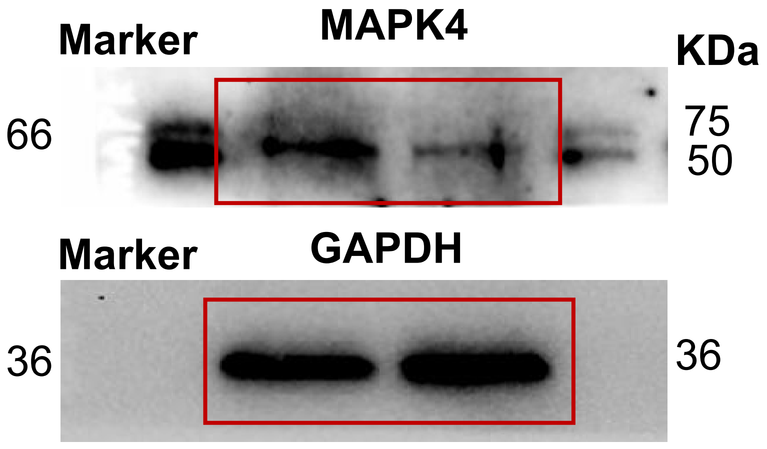

Source Fig.1K

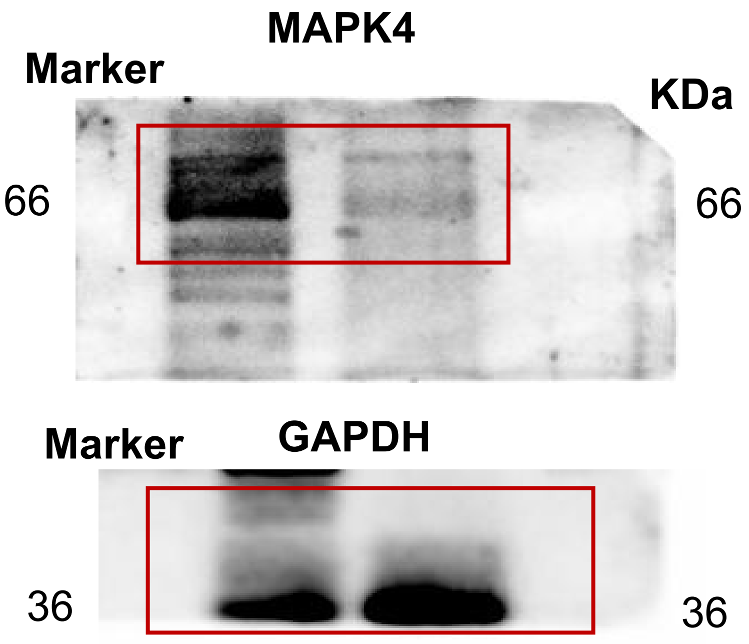

Source Fig.1L

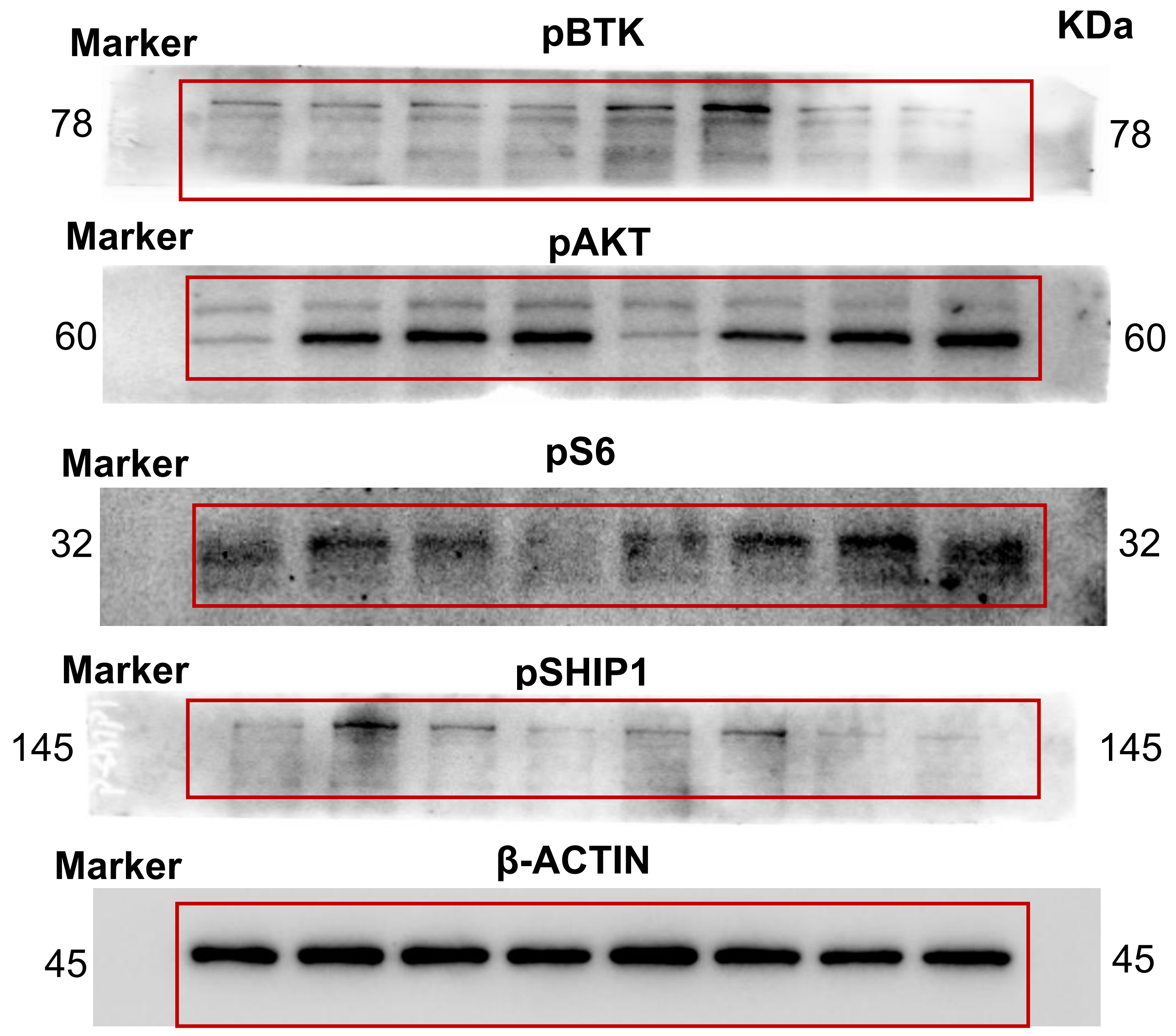

Source Fig.4D

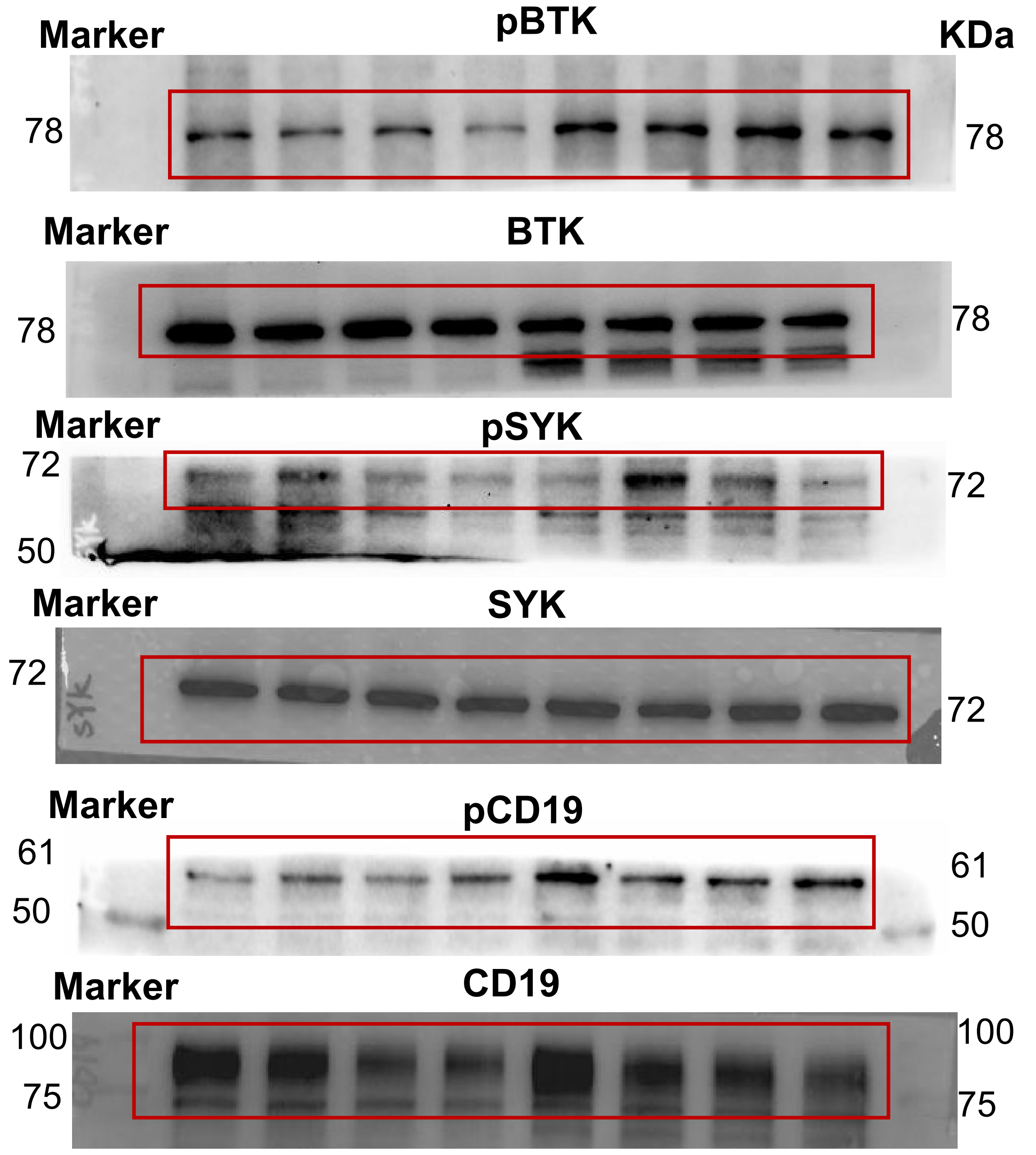

Source Fig.4H

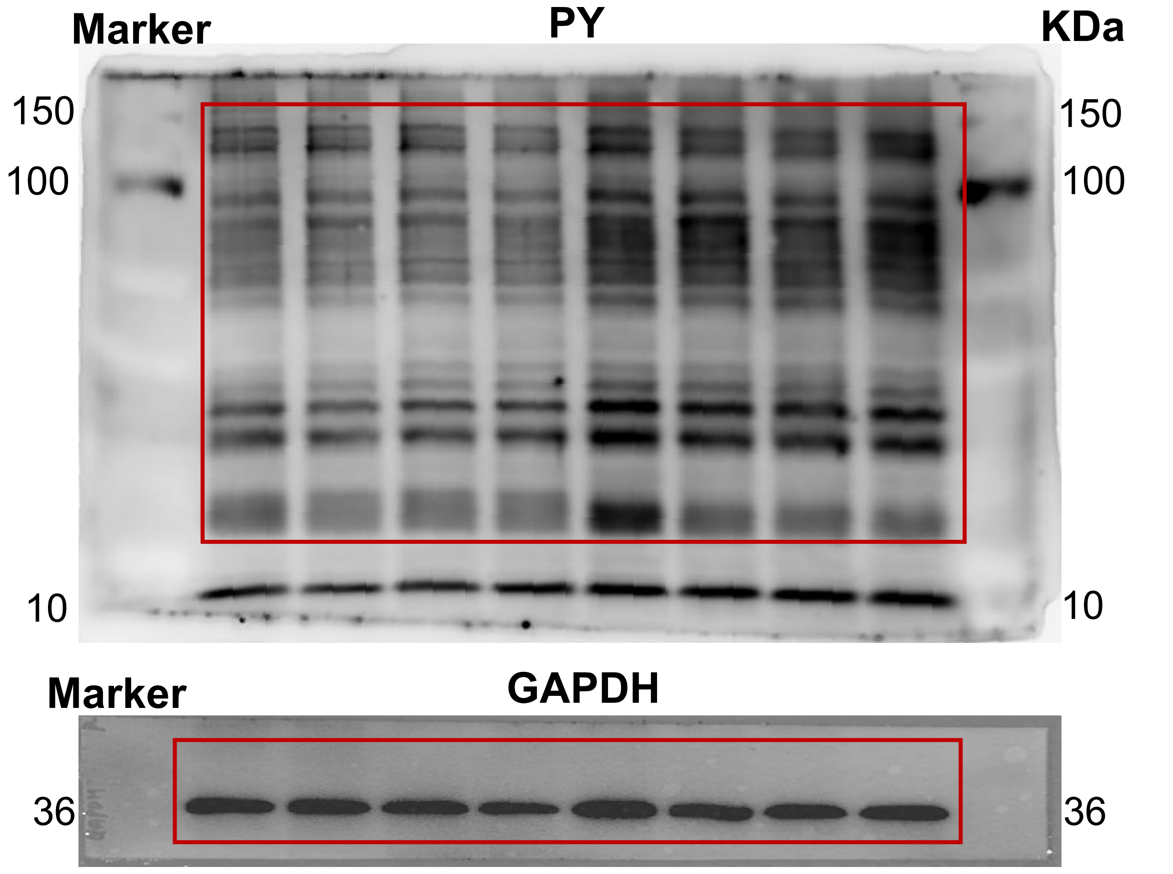

Source Fig.4T

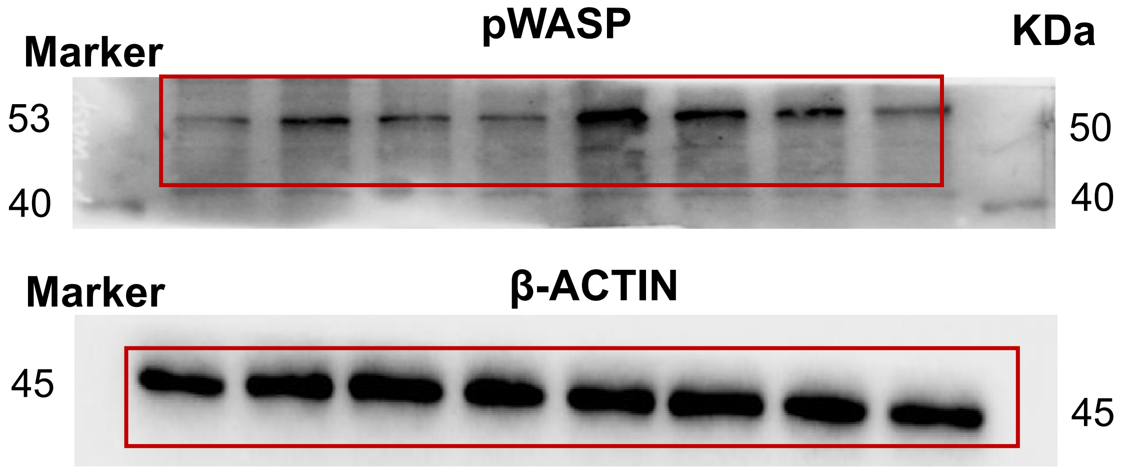

Source Fig.5D

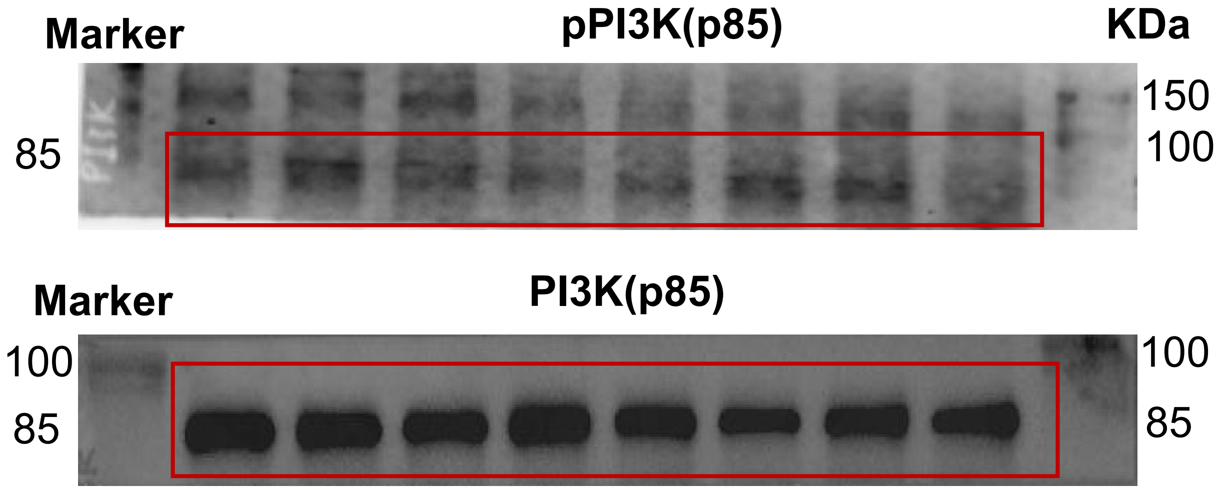

Source Fig.5E

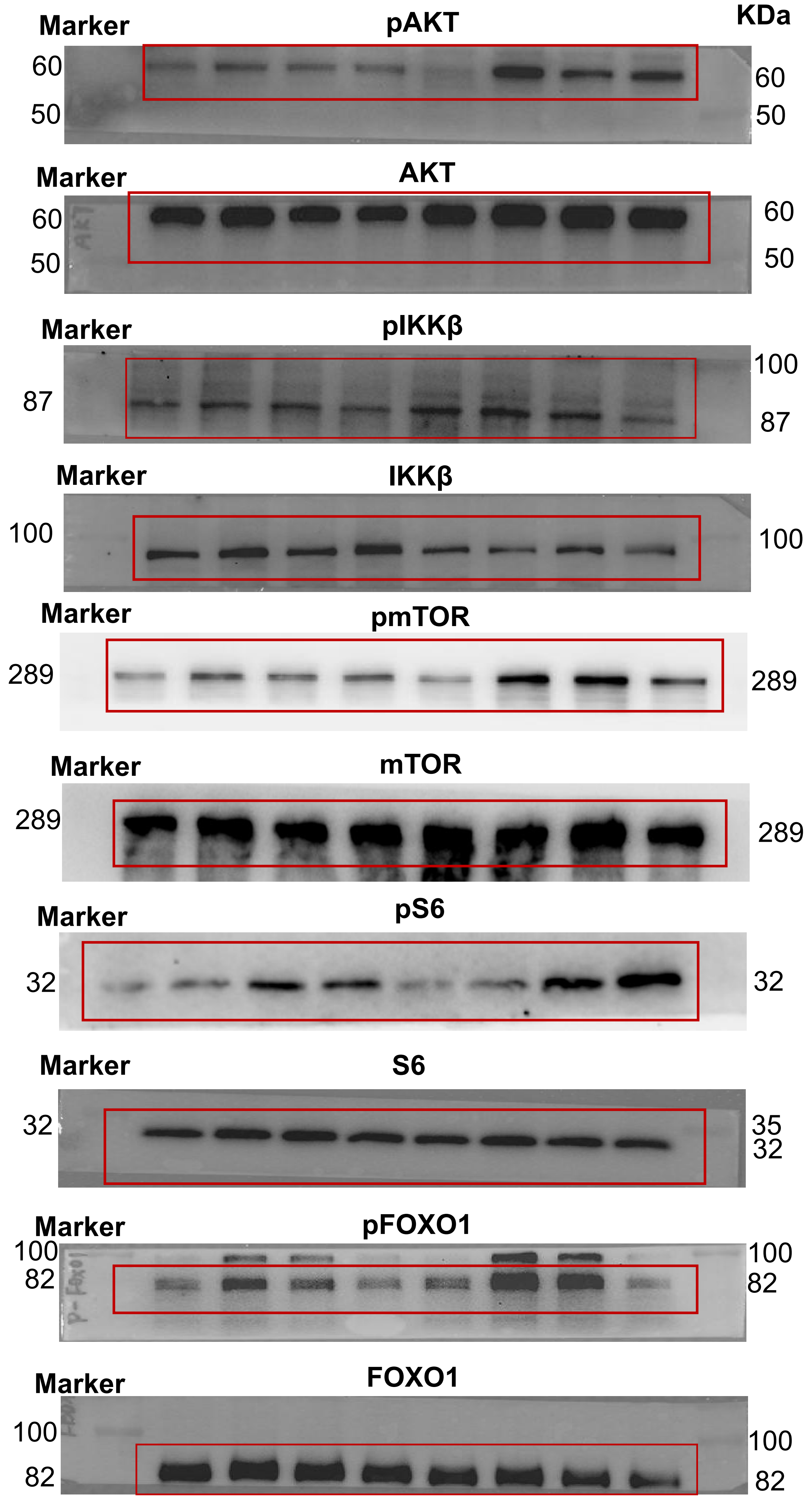

Source Fig.7A

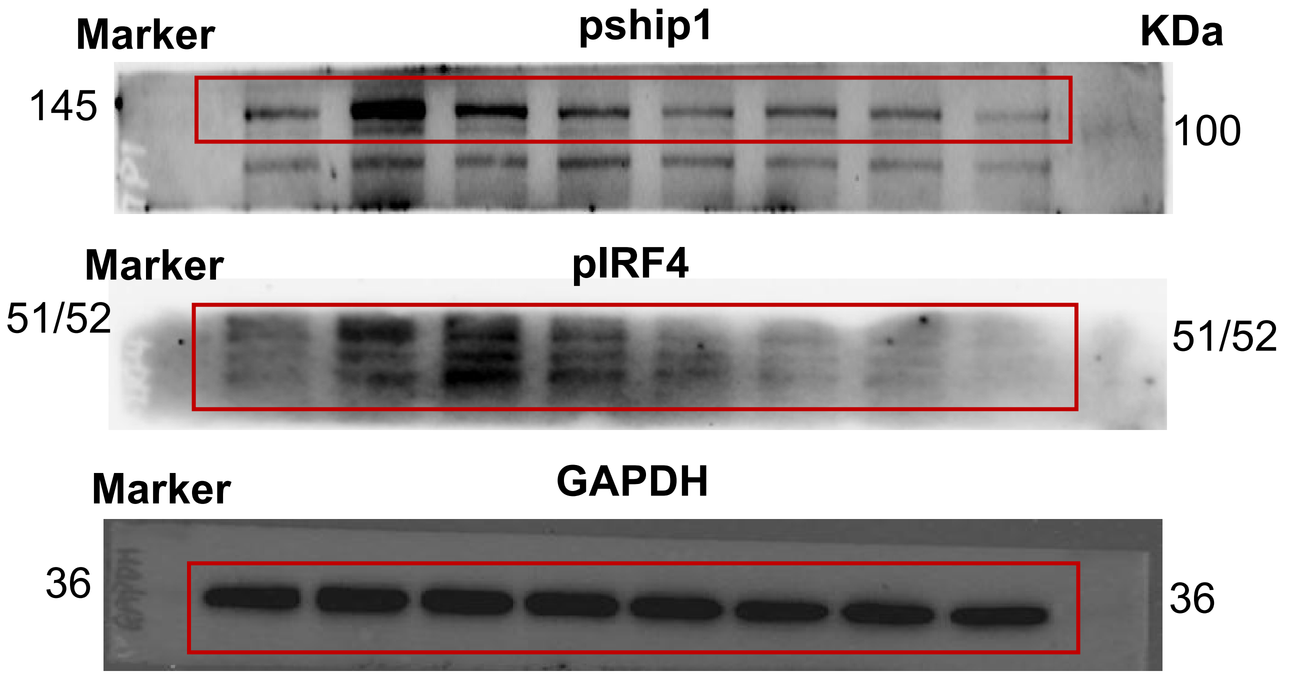

Source Fig.7C

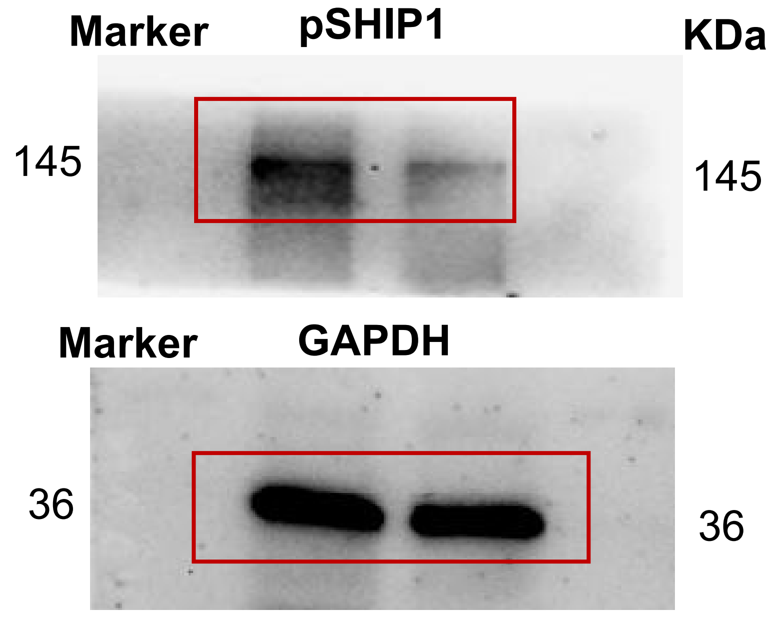

Source Fig.7D

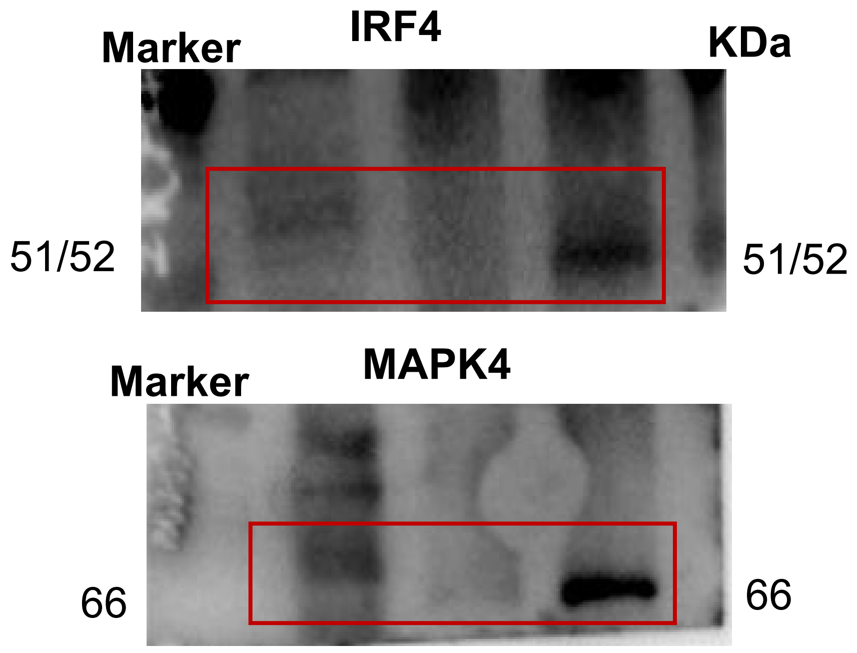

Source Fig.7E

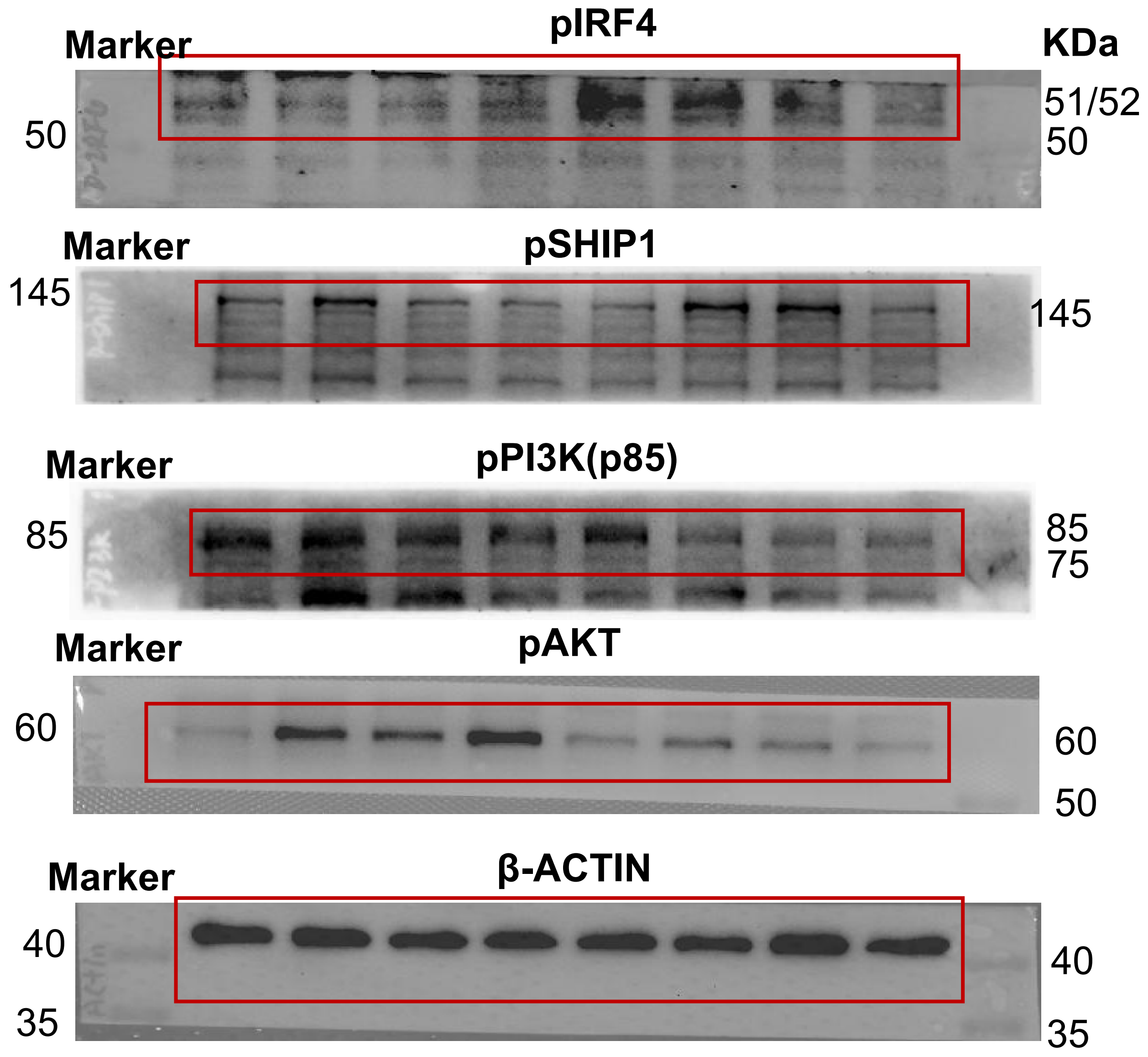

Source Fig.S1

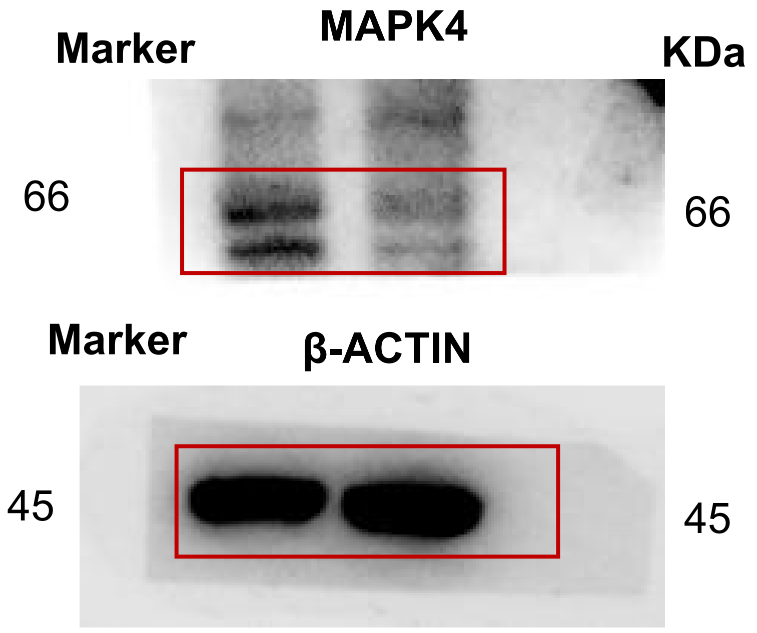

Source Fig.S4

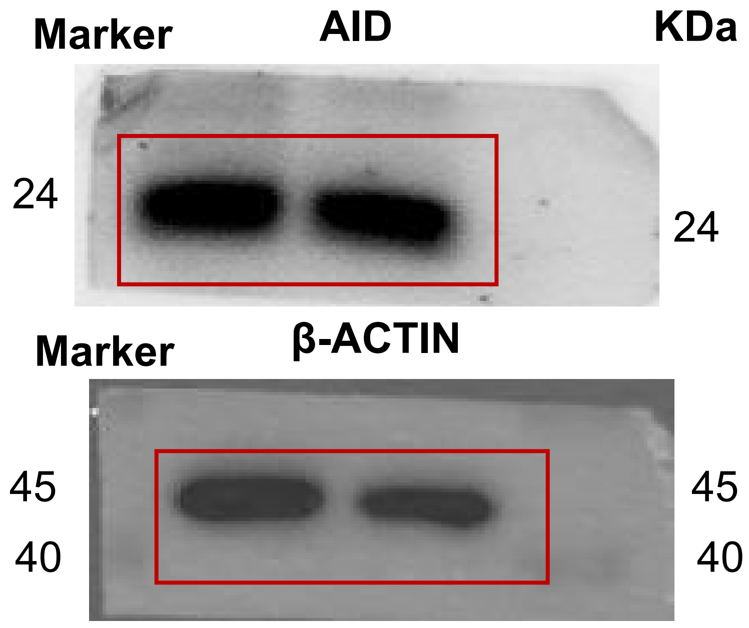

Supplement: Supplementary file 3 — Original Data File [file 41419_2025_7352_MOESM3_ESM.pdf]
